# Enhanced cell stress response and protein degradation capacity underlie artemisinin resistance in *Plasmodium falciparum*

**DOI:** 10.1128/msphere.00371-24

**Published:** 2024-10-22

**Authors:** Melissa R. Rosenthal, Sukhithasri Vijayrajratnam, Tessa M. Firestone, Caroline L. Ng

**Affiliations:** 1 Department of Pathology, Microbiology, and Immunology, University of Nebraska Medical Center, Omaha, Nebraska, USA; 2 Department of Pharmaceutical Sciences, University of Nebraska Medical Center, Omaha, Nebraska, USA; 3 Global Center for Health Security, University of Nebraska Medical Center, Omaha, Nebraska, USA; 4 Department of Biology, University of Omaha, Omaha, Nebraska, USA; Weill Cornell Medicine, New York, New York, USA

**Keywords:** *Plasmodium falciparum*, artemisinin resistance, proteostasis, unfolded protein response, ubiquitin, proteasome, proteasome-mediated protein degradation

## Abstract

Malaria remains a global health burden, killing over half a million people each year. Decreased therapeutic efficacy to artemisinin, the most efficacious antimalarial, has been detected in sub-Saharan Africa, a worrying fact given that over 90% of deaths occur on this continent. Mutations in Kelch13 are the most well-established molecular marker for artemisinin resistance, but these do not explain all artemisinin-resistant isolates. Understanding the biological underpinnings of drug resistance is key to curbing the emergence and spread of artemisinin resistance. Artemisinin-mediated non-specific alkylation leads to the accumulation of misfolded and damaged proteins and activation of the parasite unfolded protein response (UPR). In addition, the parasite proteasome is vital to artemisinin resistance, as we have previously shown that chemical inhibition of the proteasome or mutations in the β2 proteasome subunit increase parasite susceptibility to dihydroartemisinin (DHA), the active metabolite of artemisinins. Here, we investigate parasites with mutations at the Kelch13 and/or 19S and 20S proteasome subunits with regard to UPR regulation and proteasome activity in the context of artemisinin resistance. Our data show that perturbing parasite proteostasis kills parasites, early parasite UPR signaling dictates DHA survival outcomes, and DHA susceptibility correlates with impairment of proteasome-mediated protein degradation. Importantly, we show that functional proteasomes are required for artemisinin resistance in a Kelch13-independent manner, and compound-selective proteasome inhibition demonstrates why artemisinin-resistant Kelch13 mutants remain susceptible to the related antimalarial peroxide OZ439. These data provide further evidence for targeting the parasite proteasome and UPR to overcome existing artemisinin resistance.

**IMPORTANCE:**

Decreased therapeutic efficacy represents a major barrier to malaria treatment control strategies. The malaria proteasome and accompanying unfolded protein response are crucial to artemisinin resistance, revealing novel antimalarial therapeutic strategies.

## INTRODUCTION

In 2022, there were approximately 249 million cases of malaria and 608,000 deaths, of which over 90% were caused by *Plasmodium falciparum* (1). The World Health Organization recommends artemisinin-based combination therapies (ACT) as first-line treatment for uncomplicated *falciparum* malaria (1). Artemisinin resistance has been detected in Asia, South America, and Africa (1). Detection of artemisinin resistance determinants in Uganda, Rwanda, Tanzania, and the Democratic Republic of Congo (2–10) in the last few years is particularly concerning as the African continent bears the largest burden of malaria morbidity and mortality. An established molecular marker of artemisinin resistance is single point mutations in the propeller domains of Kelch13 (PF3D7_1343700) (11–13). Kelch13^C580Y^ is the predominant artemisinin resistance-associated mutation in Southeast Asia (14, 15), an area where artemisinin resistance was first reported (16, 17) and currently experiences a high prevalence of parasites that exhibit decreased ACT therapeutic efficacy (1). Since these first seminal discoveries, non-Kelch13-mediated mechanisms of artemisinin resistance have been detected both *in vitro* and *in vivo* (18–21).

Artemisinin and derivatives such as dihydroartemisinin (DHA) are bioactivated within *P. falciparum* by heme, which is a byproduct of parasite-mediated hemoglobin digestion (22–24). Kelch13^mut^ have decreased hemoglobin uptake (25, 26), decreased hemoglobin-derived digestion products (27), and decreased heme-alkylation adducts (28, 29). Together, these lead to decreased bioactivation of artemisinin. However, there are additional mechanisms beyond this that contribute to Kelch13-mediated artemisinin resistance, as evidenced by Kelch13^WT^ parasites that have a parasite clearance half-life of over 5 h, an indication of artemisinin resistance clinically (12).

Activated artemisinin and derivatives non-specifically alkylate nearby parasite proteins (30–32), causing widespread protein damage (33). In response to the DHA-mediated accumulation of misfolded proteins (34), *P. falciparum* activate the unfolded protein response (UPR) via protein kinase 4 (PK4), a *P. falciparum* ortholog of protein kinase R-like endoplasmic reticulum kinase (PERK) (33, 35, 36). PK4-mediated phosphorylation of eIF2α (p-eIF2α) attenuates global protein translation (33, 36), allowing devotion of resources to mitigate existing damage by upregulating chaperones and proteasomes. Following UPR activation as measured by the phosphorylation of eIF2α, cells including *P. falciparum* must resolve the UPR, as reflected in the dephosphorylation of eIF2α, which then allows protein synthesis to resume (37). *P. falciparum* and other Apicomplexan parasites only have components of the translational arm of the UPR (38). *P. falciparum* PK4 has been shown to be the only eIF2α kinase involved in DHA-mediated stress response (33), thereby establishing the involvement of the UPR specifically rather than the more general integrated stress response. Using the multidrug-sensitive 3D7 *P. falciparum* strain, parasites had increased survival following DHA treatment when there was either a robust phosphorylation of eIF2α or if dephosphorylation was prevented chemically (36). Prevention of eIF2α phosphorylation using GSK2606414, an inhibitor against PERK, significantly reduced parasite recrudescence following DHA treatment (36), indicating that UPR activation is key to artemisinin survival.

UPR resolution depends on proteasome-mediated degradation of misfolded and damaged proteins. Proteins targeted to the proteasome for degradation are tagged with lysine^48^ (K48)-linked ubiquitin chains, and thus accumulation of K48-linked ubiquitination is a hallmark of proteasome dysfunction. The *P. falciparum* 26S proteasome, consisting of the 19S regulatory particle (RP) and 20S core particle (CP), has been purified biochemically (39). The 19S RP binds, deubiquitinates, and unfolds target proteins (40). Focusing on the 19S mutants described in this study, Rpt4 and Rpt5 are ATPase subunits in the 19S RP base, which mediate gate opening to allow substrates into the 20S CP (41). Rpn6 acts as a scaffolding protein that stabilizes the interaction between the 19S RP and 20S CP (41). The 19S RP is important for regulating protein processing prior to proteolytic degradation within the 20S CP chamber (41). Within the 20S CP, the catalytic subunits β1, β2, and β5 display caspase-like, trypsin-like, and chymotrypsin-like activities, respectively (40). The PA28-20S CP complex and 20S CP alone have been visualized by cryo-EM (42, 43), indicating that the 20S CP exists in multiple configurations in *P. falciparum*.

The proteasome is essential for parasite survival in blood, liver, and mosquito stages (44, 45). A C31Y/F mutation in the β2 proteasome subunit compromises parasites’ survival to DHA (46), suggesting that the proteasome is critical for *P. falciparum* to survive peroxide antimalarials and act in a manner distinct from Kelch13. DHA inhibits the β5 proteasome catalytic activity of Kelch13^WT^ (33), but inhibition against other catalytic subunits and Kelch13^mut^ remains unknown. Transcriptomics and proteomics data show that Kelch13^mut^ parasites have higher levels of proteasome subunits (47, 48). Thus, we were interested in the effect of both DHA and OZ439 on all three proteolytic subunits and whether Kelch13 mutations impact DHA-mediated proteasome inhibition. Together, the UPR and ubiquitin proteasome system (UPS) function to maintain proteostasis, which reflects an equilibrium between the total amount of proteins to be degraded and the proteolytic capacity of the cell. We propose that parasite proteostasis is vital for parasite survival to DHA and other antimalarial peroxides and impacts both Kelch13- and non-Kelch13-mediated artemisinin resistance mechanisms. We and others have shown that the *P. falciparum*-specific proteasome inhibitors WLL and WLW synergize with DHA to potently kill artemisinin-resistant *P. falciparum in vitro* and *in vivo* (42, 49). These proteasome inhibitors also synergized with the DHA-related antimalarial OZ439 and the deubiquitinase inhibitor b-AP15, and was additive with the redox inhibitor methylene blue (MB), all of which are structurally diverse and possess distinct antimalarial modes of action (49). We hypothesized that these synergistic compounds further perturbed parasite proteostasis mechanisms. To interrogate the role of proteostasis mechanisms in *P. falciparum* artemisinin response and resistance, we examined UPR kinetics and proteasome activity in DHA-treated Kelch13 mutants and 19S and 20S proteasome mutants. The findings of this study highlight the importance of the *P. falciparum* UPR and proteasomes in survival to artemisinin and other antimalarial peroxides, independent of the canonical Kelch13-mediated resistance pathway.

## RESULTS

### Antimalarials synergistic with proteasome inhibitors disrupt proteostasis

**Fig 1:**
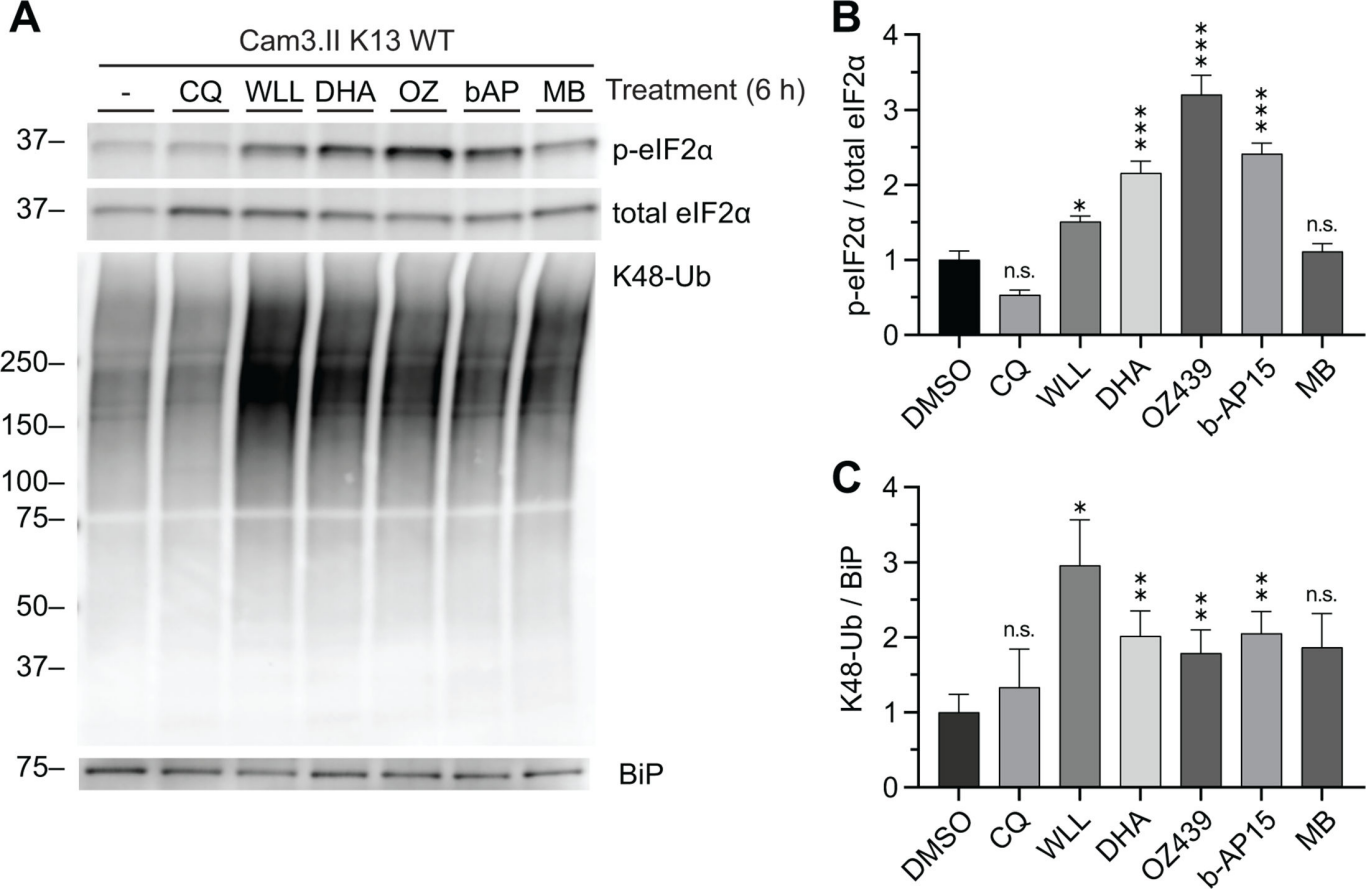
Antimalarial compounds synergistic with proteasome inhibitors disrupt proteostasis. (**A**) 26–30_hpi_ trophozoites were treated with DMSO, 50 µM CQ, 2.5 µM WLL, 50 nM DHA, 500 nM OZ439, 5 µM b-AP15, or 500 nM MB. All treatments were at 5 × IC_50_ concentrations. Western blot was performed with antibodies against p-eIF2α, total eIF2α, K48-linked ubiquitin, and BiP. Shown is a representative blot from four independent biological replicates (see Fig. S1 for replicates). (**B and C**) Densitometry analyses were performed to (**B**) assess UPR activation by normalizing p-eIF2α to total eIF2α and (**C**) assess proteasome inhibition by normalizing K48-Ub to the loading control BiP. Bar graphs indicate mean normalized integrated density ± S.E.M. Statistical significance was examined for each treatment against the DMSO control using a two-tailed paired *t*-test. *P < 0.05; **P < 0.01; ***P < 0.001; n.s. not significant.

We tested the hypothesis that antimalarials synergistic with proteasome inhibitors kill *P. falciparum* via disrupting parasite proteostasis by measuring (i) UPR activation as determined by levels of p-eIF2α normalized to total eIF2α (37) and (ii) proteasome dysfunction as determined by levels of K48-linked ubiquitination (50) normalized to the chaperone named binding immunoglobulin protein (BiP). Note that in Cam3.II strain parasites BiP does not increase in response to DHA-mediated UPR activation (48). All parasites tested herein are on a Cam3.II genetic background and either harbor Kelch13^WT^, Kelch13^R539T^, or Kelch13^C580Y^ (13), and proteasome mutants were derived on these backgrounds as described (49) (Table S1). Kelch13^WT^ trophozoites were exposed to the proteasome inhibitor WLL, three synergistic compounds with WLL: (i) dihydroartemisinin (DHA), (ii) OZ439, and (iii) b-AP15, a compound additive with WLL: methylene blue (MB), and a compound antagonstic with WLL: chloroquine (CQ) (49), as well as the control solvent dimethyl sulfoxide (DMSO). All synergistic compounds tested resulted in UPR activation and accumulation of K48-linked ubiquitination (Fig. 1). OZ439 yielded the highest level of UPR activation followed by DHA and b-AP15, and then the proteasome inhibitor WLL (Fig. 1A and B; Fig. S1). WLL-treated parasites showed the highest levels of K48-linked ubiquitination as expected (Fig. 1A and C; Fig. S1), indicating that the observed WLL-mediated UPR activation is likely a secondary effect (51, 52). MB did not lead to significant UPR activation or K48-linked ubiquitination (Fig. 1A through C; Fig. S1). In contrast, parasites treated with the antagonistic compound CQ (49) showed no difference in UPR activation or K48-linked ubiquitination compared to DMSO-treated parasites (Fig. 1A through C; Fig. S1). Together, these data indicate that compounds that synergize with proteasome inhibitors to potently kill malaria parasites also disrupt proteostasis.

### UPR regulation in artemisinin-sensitive vs artemisinin-resistant parasites

**Fig 2:**
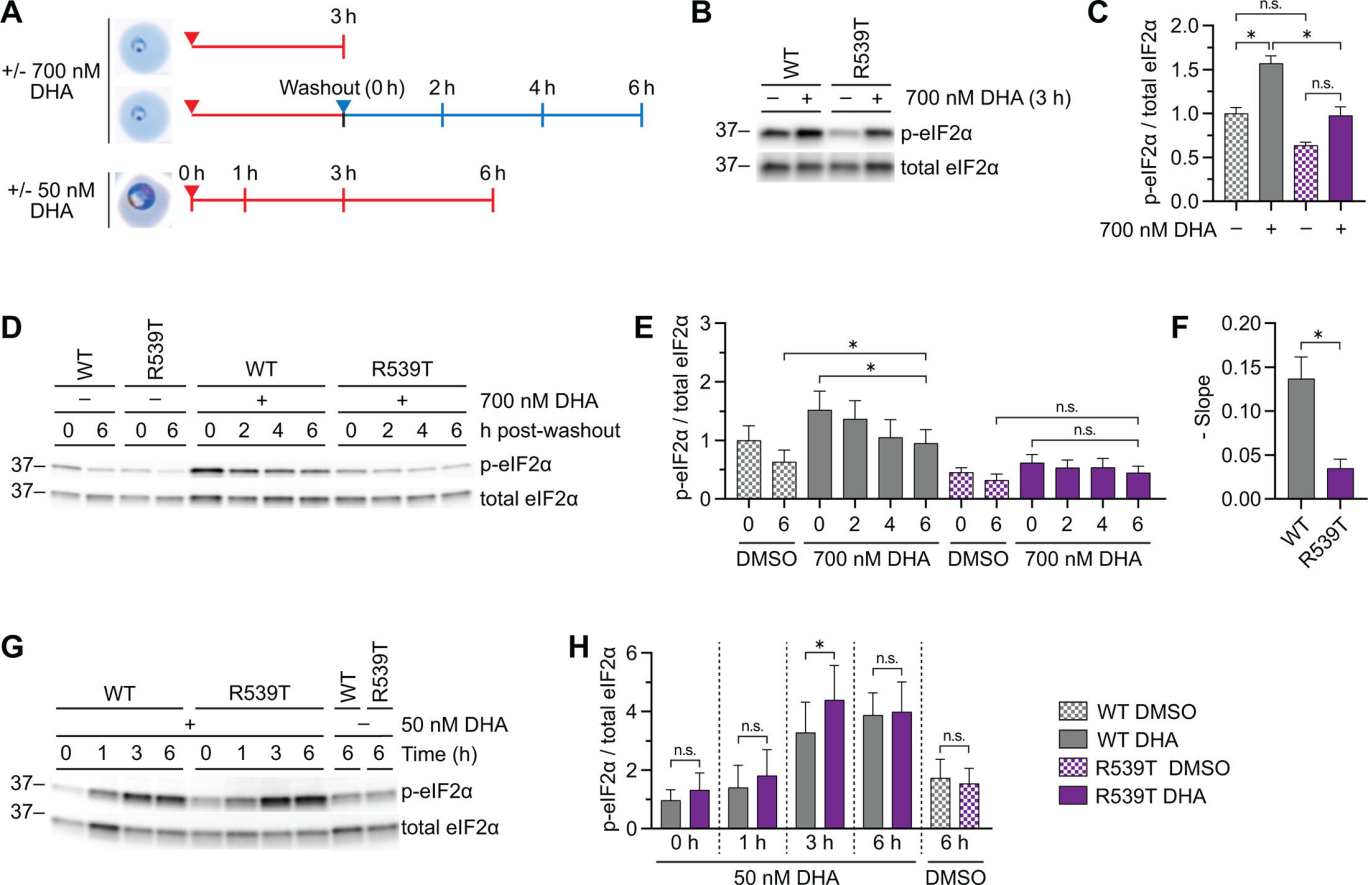
Kelch13^WT^ and Kelch13^mut^ parasites differentially regulate the UPR. (**A**) Diagram of experiments. Red arrows indicate the addition of the drug, and red lines indicate drug treatment. The blue triangle indicates the removal of the drug, and the blue line indicates the time after the washout. (**B**) DMSO- or DHA-treated Kelch13^WT^ and Kelch13^R539T^ 0–3_hpi_ rings subject to Western blot with antibodies against p-eIF2α and total eIF2α. Shown is a representative blot from three independent biological replicates (see Fig. S2A for replicates). (**C**) Densitometry analyses to determine UPR activation. (**D**) Kelch13^WT^ and Kelch13^R539T^ 0–3_hpi_ rings were treated with DMSO or 700 nM DHA for 3 h. Drug was washed off and parasites were harvested at the indicated times and subjected to Western blot. Representative blot shown from three independent biological replicates (see Fig. S2B for replicates). (**E**) Densitometry analyses to assess UPR activation. (**F**) Rate of de-phosphorylation of eIF2α following drug removal was calculated over time, and the mean negative slope ± S.E.M. was plotted. (**G**) Kelch13^WT^ and Kelch13^R539T^ 26–30_hpi_ trophozoites were treated with DMSO or 50 nM DHA for indicated times and subject to Western blot. Representative blot shown from four independent biological replicates (see Fig. S2F for replicates). (**H**) Densitometry analyses to determine UPR activation. Bar graphs indicate mean normalized integrated density ± S.E.M. from at least three independent biological replicates. Statistical significance was examined for the indicated comparisons using a two-tailed paired *t*-test. *P < 0.05, n.s. not significant.

To delve deeper, we examined the kinetics of UPR activation and resolution in artemisinin-sensitive and artemisinin-resistant parasites. Kelch13^WT^ and Kelch13^R539T^ rings were treated with the physiologically relevant concentration of 700 nM DHA for 3 h, mimicking conditions of the ring-stage survival assay (RSA) used to delineate artemisinin resistance *in vitro* (49, 53, 54) (Fig. 2A, top and middle panels). In untreated parasites, Kelch13^WT^ displayed basal levels of a slight but statistically insignificant increase in UPR activation compared to Kelch13^R539T^. In response to DHA, Kelch13^WT^ had significantly elevated levels of p-eIF2α when compared to the DMSO-treated counterpart as well as DHA-treated Kelch13^R539T^ (Fig. 2B and C; Fig. S2A). Together, the data show that compared to Kelch13^R539T^, Kelch13^WT^ have slightly elevated UPR at basal levels in the absence of DHA, and the UPR is significantly heightened upon exposure to DHA.

Next, UPR resolution was monitored in these parasites following drug removal. Levels of p-eIF2α declined over time in both parasites following DHA washout (Fig. 2D through F; Fig. S2B). At 6 h post-washout, in contrast to Kelch13^R539T^ whose UPR activation returned to basal levels, levels of p-eIF2α in Kelch13^WT^ remained elevated relative to the DMSO-treated control (Fig. 2D and E), demonstrating an inability to resolve the UPR and suggests a prolonged state of stress. Kelch13^C580Y^, which display an intermediate RSA value between Kelch13^WT^ and Kelch13^R539T^ (13, 46), displayed intermediate UPR activation and resolution as measured by the rate of p-eIF2α de-phosphorylation following DHA removal (Fig. S2C through E). Interestingly, Kelch13^C580Y^ β2^C31Y^, which have increased sensitivity to DHA (46), also had elevated levels of p-eIF2α at 6 h post-washout compared to DMSO-treated counterparts (Fig. S2C through E). Together, the data suggest that parasites sensitized to DHA are unable to resolve DHA-mediated UPR activation despite removal of the drug.

We then examined Kelch13^WT^ and Kelch13^R539T^ trophozoites (Fig. 2A, bottom panel). Parasites responded to DHA with UPR activation in a time-dependent manner. At 3 h post-treatment, Kelch13^R539T^ had greater UPR activation compared to Kelch13^WT^ but leveled out at 6 h (Fig. 2G and H; Fig. S2F), suggesting a fine-tuned robust UPR activation in Kelch13^R539T^ trophozoites. These data show that the kinetics of UPR activation and resolution are dependent on both the Kelch13 genotype and the parasite intraerythrocytic developmental stage. As there is a feedback loop between UPR activation and proteasome activity (51, 52), we next sought to determine proteasome activity in parasites that differed in genotype at Kelch13 and/or proteasome catalytic subunits in the absence or presence of DHA and related antimalarials.

### DHA and OZ439 inhibit parasite proteasome activity

Proteasome activity in DHA-treated Kelch13^WT^, Kelch13^R539T^, and Kelch13^C580Y^ was examined at the trophozoite stages when the UPS is upregulated (55, 56) and because artemisinin treatment does not produce a detectable increase in ubiquitination at the early ring stage (57). The proteolytic activity of proteasome catalytic subunits was examined in DHA- and OZ439-treated trophozoites using the fluorogenic peptidyl substrates Ac-nLPnLD-AMC, Ac-RLR-AMC, or Suc-LLVY-AMC to assess β1, β2, and β5, respectively (58). WLL was used as a positive control for the inhibition of β2 and β5 activity (42). No known inhibitor of plasmodial β1 exists, though high concentrations of WLL moderately inhibit plasmodial β1 activity (42).

**Fig 3:**
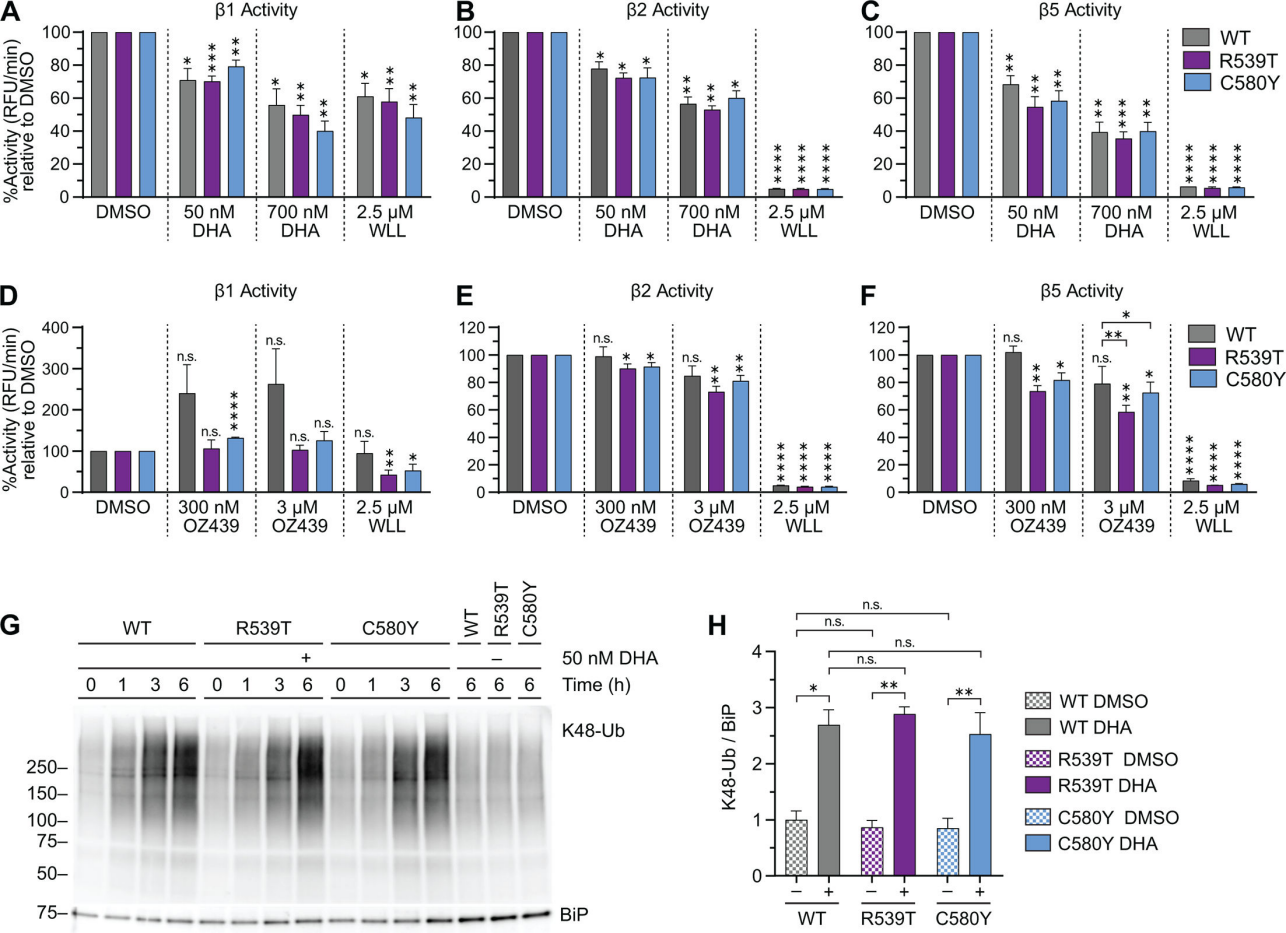
Antimalarial peroxides DHA and OZ439 inhibit parasite proteasome activity. (**A–C**) Trophozoites were treated with DMSO or indicated drugs for 3 h, then lysates were incubated with (**A**) Ac-nLPnLD-AMC, (**B**) Ac-RLR-AMC, or (**C**) Suc-LLVY-AMC to assess β1, β2, and β5 activity, respectively. Fluorescence was plotted over time and the percentage of activity was quantified by calculating the slope of the line and normalizing to the slope of DMSO-treated parasites. Bar graphs indicate the mean percentage of activity ± S.E.M. from three independent biological replicates, each consisting of two technical replicates. A two-tailed Student’s *t*-test was performed between DMSO and drug-treated counterparts, and statistical significance is indicated above the bars as vertical asterisks. Comparisons were also performed between Kelch13^WT^ and Kelch13^mut^ for each treatment condition, but only significant comparisons are denoted. (**D–F**) Trophozoites were treated with DMSO or indicated drugs for 3 h, then proteasome activity was assessed as described above. Three independent biological replicates were performed, each consisting of two technical replicates. Statistical analyses were performed as described above. (**G**) Trophozoites treated with DMSO or 50 nM DHA for the indicated times were subject to Western blot with antibodies against K48-linked ubiquitin and BiP. Shown is a representative blot of four independent biological replicates (see Fig. S3 for replicates). (**H**) Densitometry analyses to determine proteasome inhibition. Bar graphs indicate mean normalized integrated density ± S.E.M. A two-tailed paired *t*-test was performed for the indicated comparisons. *P < 0.05; **P < 0.01; ***P < 0.001; ****P < 0.0001; n.s. not significant.

Extending previous observations (33), DHA inhibited all three catalytic subunits of proteasomes derived from Kelch13^WT^, Kelch13^R539T^, and Kelch13^C580Y^ trophozoites in a statistically significant and concentration-dependent manner (Fig. 3A through C). No significant difference was detected in the catalytic inhibition of proteasomes derived from Kelch13^WT^ vs Kelch13^mut^ parasites. The effect of OZ439 on proteasomes was also tested (Fig. 3D through F). Interestingly, at the physiologically relevant peak plasma concentration of 3 µM OZ439 (59), we observed selective inhibition of β5 activity in Kelch13^mut^ (Fig. 3F). In concordance with the proteolytic assay, we saw an accumulation of K48-linked ubiquitination in response to DHA treatment in a time-dependent manner regardless of Kelch13 genotype (Fig. 3G and H; Fig. S3A and B). Collectively, these data show that DHA equally inhibits parasite proteasomes regardless of Kelch13 genotype and that OZ439 selectively inhibits the β5 catalytic activity of Kelch13^R539T^ and Kelch13^C580Y^.

### Mutations in 19S RP subunits increase parasite susceptibility to DHA

**Fig 4:**
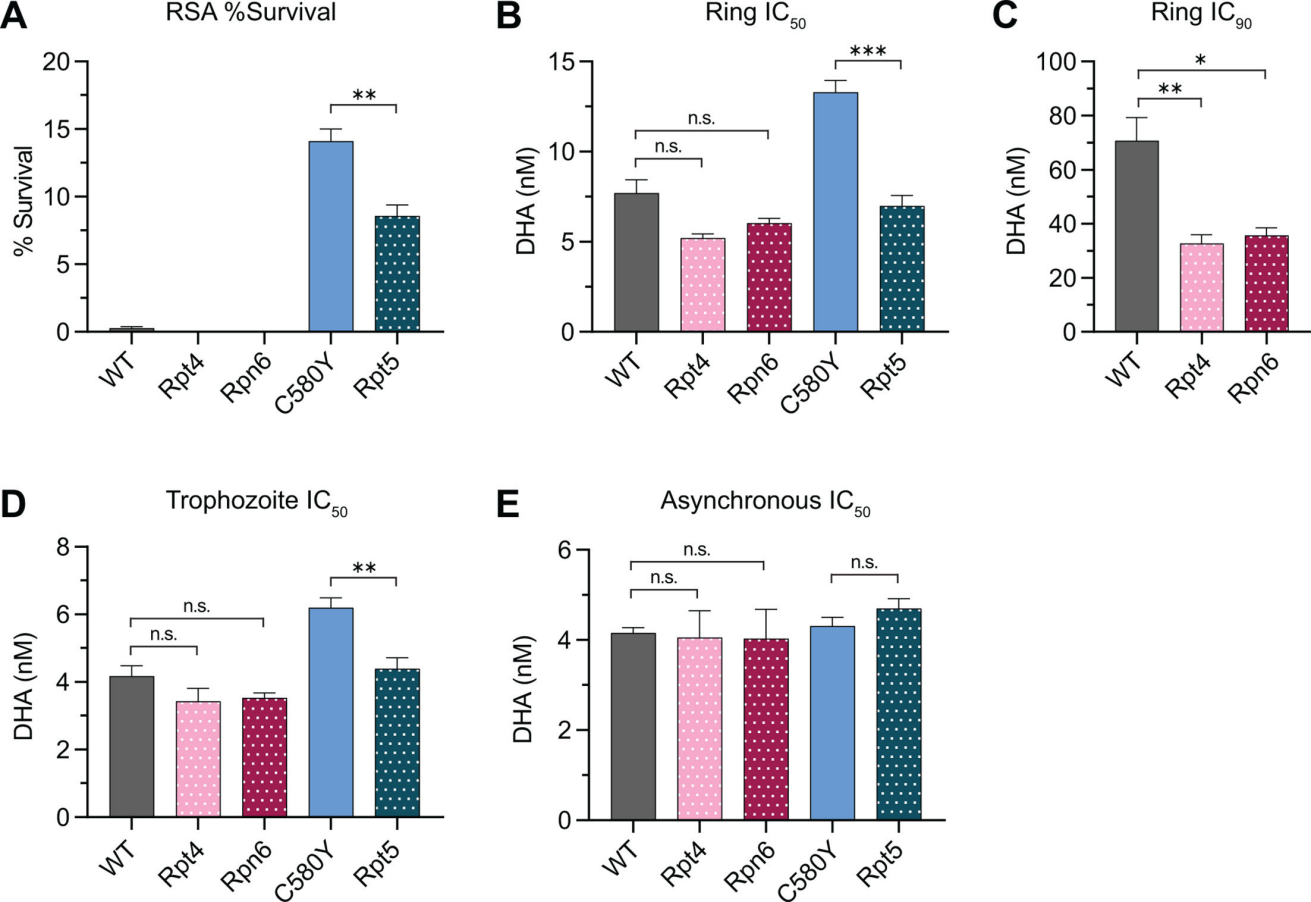
19S RP proteasome mutants display increased susceptibility to DHA. (**A–C**) Indicated parasites subjected to RSA and ring-stage dose-response assays. Six independent biological replicates were performed. Dose-response curves are in Fig. S4A. (**A**) Percentage of survival in RSA. (**B**) Ring-stage IC_50_ and (**C**) ring-stage IC_90_ values were determined using non-linear regression analysis. (**D**) Dose-response assays conducted on 26–30_hpi_ trophozoites. Seven independent biological replicates were performed. Dose-response curves are in Fig. S4B. (**E**) Dose-response assays conducted on asynchronous parasites in five independent biological experiments. Dose-response curves are in Fig. S4C. Bar graphs show mean IC_50_ or IC_90_ values ± S.E.M. Statistical significance was examined for each proteasome mutant against the cognate parental strain using a Mann-Whitney *U* test. *P < 0.05; **P < 0.01; ***P < 0.001; n.s. not significant.

To determine the impact of 19S RP and Kelch13 mutations on parasite survival to DHA, we performed RSA and ring-stage growth inhibition assays using Cam3.II strain parasites with Kelch13^WT^ that harbored either Rpt4^E380*^ or Rpn6^E266K^, and Kelch13^C580Y^ harboring Rpt5^G319S^ (49) (Fig. S4A; Table 1; Table S1). As expected, all parasites harboring Kelch13^WT^ regardless of proteasome mutations displayed RSA values < 1%, indicating artemisinin sensitivity (53) (Fig. 4A). Compared to the parental Kelch13^WT^, Rpt4^E380*^and Rpn6^E266K^ were sensitized to DHA displaying decreased ring-stage IC_50_ and IC_90_ values (Fig. 4B and C) and demonstrated correspondingly steeper dose-response curves (Fig. S4A). No differences in IC_50_ values between parental and proteasome mutant strains were detected in trophozoites or asynchronous parasites (Fig. 4D and E; Fig. S4B and C).

**TABLE 1:** Characterization of DHA resistance in Cam3.II strain parasites with Kelch13 and/or proteasome subunit mutations^*a*^

Parasite strain	RSA (%)	Ring IC_50_ (nM)	Ring IC_90_ (nM)	Trophozoite IC_50_ (nM)	Asynchronous IC_50_ (nM)
Cam3.II Kelch13^WT^	0.3	7.7 ± 0.7	70.7 ± 8.7	4.2 ± 0.3	4.2 ± 0.1
Cam3.II Kelch13^WT^ Rpt4^E380*^	0.0	5.2 ± 0.2	32.7 ± 3.2	3.4 ± 0.4	4.1 ± 0.6
Cam3.II Kelch13^WT^ Rpn6^E266K^	0.0	6.0 ± 0.3	35.6 ± 2.9	3.5 ± 0.1	4.0 ± 0.6
Cam3.II Kelch13^C580Y^	14.1	13.3 ± 0.7	78.1 ± 7.0	6.2 ± 0.3	0.2 ± 0.2
Cam3.II Kelch13^C580Y^ Rpt5^G319S^	8.6	7.0 ± 0.6	51.5 ± 6.0	4.4 ± 0.3	4.7 ± 0.2

^*a*^ Ring-stage survival assay, IC_50_, and IC_90_ values of dihydroartemisinin tested at the 0–3_hpi_ ring stages, 26-30_hpi_ trophozoite stages, or with mixed stage (asynchronous) parasites. Mean values ± S.E.M.

Rpt5^G319S^ had approximately twofold lower RSA, ring IC_50_, and trophozoite IC_50_ values compared to parental Kelch13^C580Y^ (Fig. 4A, B, and D; Fig. S4A and B). These differences were not observed in asynchronous cultures (Fig. 4E; Fig. S4C). These data demonstrate that mutations in 19S RP subunits increase parasite susceptibility to DHA regardless of Kelch13 genotype.

### Parasites with antimalarial peroxide susceptibility have impaired proteasome-mediated protein degradation

**Fig 5:**
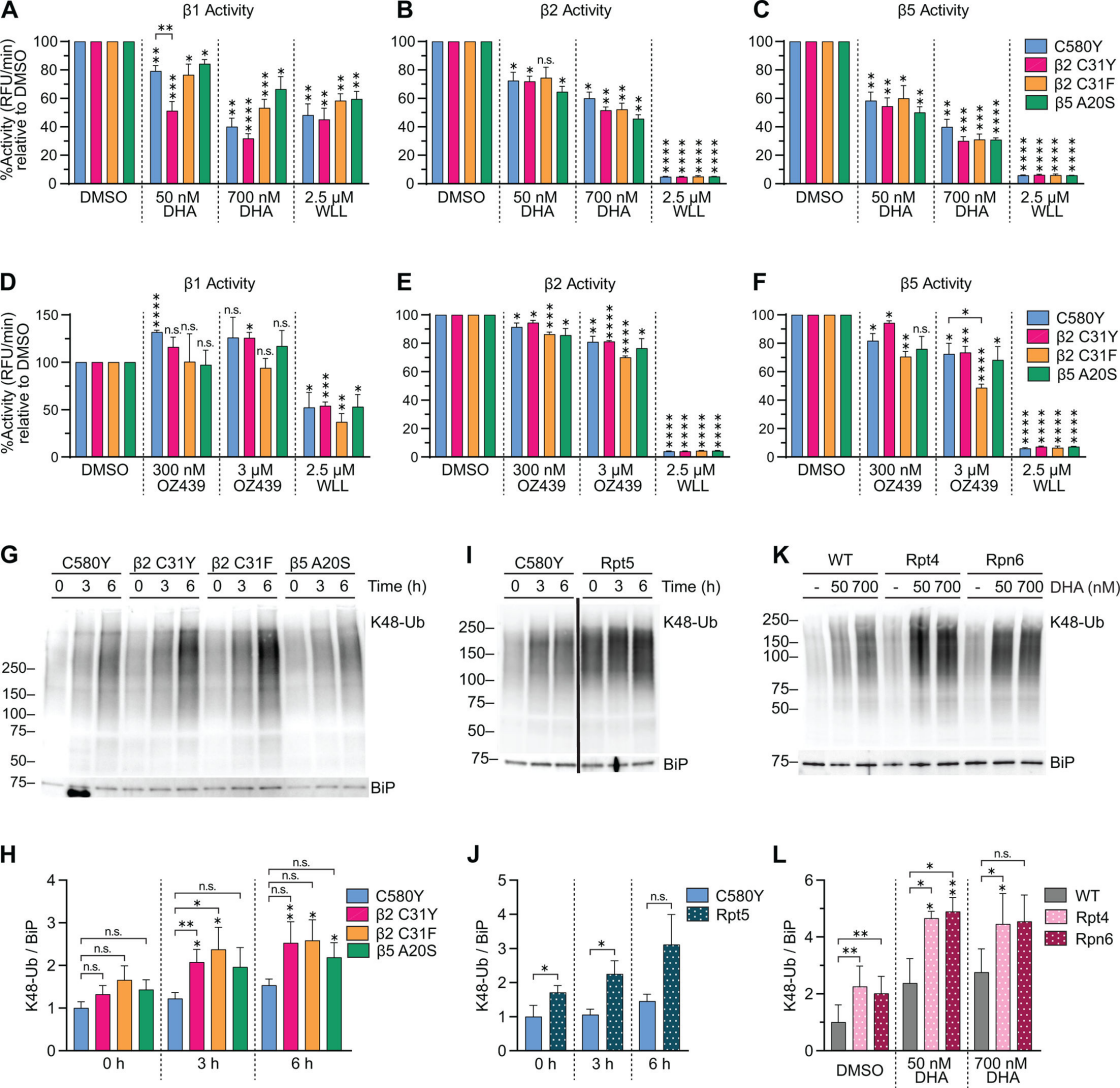
Proteolytic activity of proteasome mutants. (**A–C**) Trophozoites were treated with DMSO or indicated drugs for 3 h, then lysates were incubated with (**A**) Ac-nLPnLD-AMC, (**B**) Ac-RLR-AMC, or (**C**) Suc-LLVY-AMC to assess β1, β2, and β5 activity, respectively. Proteasome activity was assessed as described in Fig. 3. Three independent biological replicates, each consisting of two technical replicates, were performed. A two-tailed Student’s *t*-test was performed between DMSO- and drug-treated counterparts, indicated above the bars as vertical asterisks. Comparisons were also performed between proteasome mutants and Kelch13^C580Y^ and only significant comparisons are denoted with brackets. (**D–F**) Trophozoites were treated with DMSO or indicated drugs for 3 h, then proteasome activity was assessed as described. Three independent biological replicates, each consisting of two technical replicates, were performed. Statistical analyses were performed as described above. (**G**) Trophozoites were treated with 50 nM DHA for the indicated times, then Western blot was performed with antibodies against K48-linked ubiquitin and BiP. Representative blot is shown out of five independent experiments (see Fig. S5A for replicates). (**H**) Densitometry analyses were performed to assess proteasome activity. Bar graph indicates mean normalized integrated density ± S.E.M. A two-tailed paired *t*-test was performed to compare treated and untreated counterparts, indicated above the bars as vertical asterisks. A two-tailed paired *t*-test was also performed between proteasome mutants and Kelch13^C580Y^, indicated by brackets. (**I**) Trophozoites were treated with 50 nM DHA for indicated times, then subjected to Western blot. Shown is a representative blot out of four independent experiments (see Fig. S5B for replicates). (**J**) Densitometry analyses. (**K**) Trophozoites were treated with DMSO, 50 nM, or 700 nM DHA for 3 h and then Western blot was performed. Shown is a representative blot out of three independent experiments (see Fig. S5C for replicates). (**L**) Densitometry analyses. *P < 0.05; **P < 0.01; ***P < 0.001; ****P < 0.0001; n.s. not significant.

Proteasome proteolytic activity of DHA- and OZ439-treated trophozoites derived from Cam3.II Kelch13^C580Y^ parasites that harbored β2^C31Y^, β2^C31F^, or β5^A20S^ was examined (Fig. S1). DHA significantly inhibited all catalytic subunits in all parasites tested in a concentration-dependent manner (Fig. 5A through C). DHA inhibited β1 activity of β2^C31Y^ to a greater extent compared to the parental strain (Fig. 5A). OZ439 did not inhibit β1 activity (Fig. 5D) but did inhibit β2 and β5 activities in all parasites tested (Fig. 5E and F). Note that 3 µM OZ439 inhibited the β5 catalytic site of β2^C31F^ by 52% compared to only 28% in Kelch13^C580Y^ (Fig. 5F), and this correlates with increased susceptibility to OZ439 in the β2 mutant (46). No other significant difference in catalytic inhibition was observed between Kelch13^C580Y^ and 20S CP mutants treated with DHA or OZ439.

Next, inhibition of proteasome-mediated protein degradation was evaluated in 20S CP (Fig. 5G and H) and 19S RP mutants (Fig. 5I through L) by assessing the accumulation of K48-linked ubiquitination. Intriguingly, DHA-treated β2^C31Y^ and β2^C31F^ had approximately twofold higher levels of K48-linked ubiquitination in response to 3-h exposure to DHA compared to Kelch13^C580Y^ (Fig. 5G and H; Fig. S5A). β5^A20S^, which did not display altered sensitivity to DHA or OZ439 (46), had minor and statistically insignificant increases in ubiquitination. A statistically significant twofold increase in K48-linked ubiquitination was also observed in Rpt5^G319S^, Rpt4^E380*^, and Rpn6^E266K^ compared to their respective parental strains (Fig. 5I through L; Fig. S5B and C).

Since we observed that the UPR was differentially activated in Kelch13^WT^ vs Kelch13^mut^ parasites, we were interested in determining UPR activation kinetics in proteasome mutants. However, no significant difference in UPR activation was observed between parental and proteasome mutant parasites at the early ring (Fig. S2C through E) or trophozoite stages (Fig. S6). We note that in trophozoite stages, Kelch13^C580Y^, β2^C31Y^, β2^C31F^, β5^A20S^, and Rpt5^G319S^ significantly induced UPR activation compared to untreated counterparts, but no difference in UPR activation was observed between Kelch13^C580Y^ and proteasome mutants (Fig. S6). Collectively, these data indicate that a defect in proteasome-mediated protein degradation underlies the heightened sensitivity of proteasome mutants to antimalarial peroxides and that this defect is not mediated by increased inhibition of proteasome catalytic subunits.

## DISCUSSION

As the prevalence of artemisinin resistance continues to rise, it becomes increasingly urgent to delineate a mechanism of resistance to inform future drug discovery and implementation of antimalarial combination therapies. In addition to the widespread artemisinin resistance in the Southeast Asian region, recent reports of Kelch13-mediated artemisinin resistance in the African region are of particular concern (2–10). We have previously shown that proteasome inhibitors effectively kill artemisinin-resistant parasites and strongly synergize with DHA (49, 60). In addition, parasites moderately resistant to proteasome inhibitors are sensitized to DHA (46). Importantly, proteasome inhibitors are effective against Ugandan parasite isolates (61).

Exploration of the kinetics of UPR activation and resolution as well as proteasome activity in DHA-treated parasites differing in the loci of Kelch13 or proteasome subunits yielded some surprising results. First, the data confirmed our hypothesis that antimalarials synergistic with proteasome inhibitors such as DHA, OZ439, and b-AP15 perturb proteostasis by upregulating the UPR and inhibiting proteasome-mediated protein degradation. In contrast, antimalarials that antagonize with proteasome inhibitors such as CQ had no effect on these measurements of proteostasis perturbations. MB, which was additive with proteasome inhibitors at the trophozoite stages (49), had an intermediate increase in UPR activation and ubiquitination. These data suggest that directly interfering with proteostasis mechanisms is a promising antimalarial therapeutic strategy.

Second, we found that early parasite responses to DHA dictate eventual survival outcomes. Transcriptomics and proteomics data point to a role for Kelch13^mut^ in broadly enhancing the parasite’s stress response (47, 48). Here, we show that Kelch13^WT^ hyperactivate the UPR at early ring stages, indicating that these parasites are either (i) experiencing increased levels of stress and/or (ii) the UPR is dysfunctionally regulated. Kelch13^mut^ have reduced hemoglobin uptake and digestion and consequently reduced artemisinin activation, and the role of Kelch13 in hemoglobin uptake appears to be restricted to the ring stage (26, 27). Accordingly, it would be expected that the misfolded protein load in Kelch13^mut^ would be lower and less prone to trigger the UPR at the ring stage. This hypothesis is consistent with our observations that early ring-stage Kelch13^R539T^ display little UPR activation in response to DHA and dephosphorylate eIF2α following drug removal. In contrast, DHA-treated Kelch13^WT^ early rings display hyperactivation of the UPR and are unable to resolve the UPR as seen by residual eIF2α phosphorylation 6 h after DHA removal. These data are also consistent with findings that following a 3-h pulse with 700 nM DHA on ring-stage parasites, Kelch13^R539T^ parasites begin to resume protein turnover as early as 9 h after drug removal while Kelch13^WT^ do not, and the differences become more pronounced at 15 h post-drug removal (27). A halt in protein translation at the early ring stages leads to deleterious effects, likely due to a lack of proteins important in parasite development and survival. In contrast, at the trophozoite stages where hemoglobin digestion is increased (62) and Kelch13 is not involved in hemoglobin uptake (26), we found that the UPR was activated earlier in DHA-treated Kelch13^R539T^ parasites. A more robust UPR activation at the trophozoite stages could be advantageous to Kelch13^mut^, giving them a jumpstart on mitigating protein damage, given that metabolic processes and protein abundance are greatly increased during these intraerythrocytic stages (27).

Previous studies demonstrate conflicting data regarding UPR activation in the early ring stages of Kelch13^WT^ vs Kelch13^mut^. Consistent with what we observed, Dd2 Kelch13^WT^ 0–3_hpi_ rings treated with 700 nM DHA for 15 min displayed more robust UPR activation than Dd2 Kelch13^C580Y^ 0–3_hpi_ rings (36). However, the authors observed that Kelch13^mut^ displayed elevated basal UPR activation, which contrasts with our observation (36). This disparity could be attributed to differences in the genetic backgrounds of the parasites examined. Dd2 was adapted to the laboratory in the 1970s prior to widespread artemisinin usage, while Cam3.II was adapted in the 2010s and is an artemisinin-resistant isolate. In a separate study, it was observed that relative to Cam3.II Kelch13^WT^ 0–8_hpi_ rings, Cam3.II Kelch13^R539T^ had elevated UPR activation under basal conditions and in response to a 3-h treatment with 700 nM DHA (27). It is possible that differences between early- and mid-ring stages could explain discrepancies between these data.

Kelch13^mut^ and artemisinin-resistant clinical isolates have increased levels of proteasome subunits by transcriptomics and proteomics (47, 48). However, we observed no difference in proteasome activity between isogenic Kelch13^WT^ vs Kelch13^mut^ at basal levels or when DHA-treated as assessed by peptidyl substrate cleavage or protein degradation. Since the proteasome is a multi-subunit complex, upregulation of some proteasome subunits may be insufficient to modulate proteasome activity. It is also possible that the assays used here are unable to detect slight differences in proteasome activity, which may be biologically relevant. Collectively, these data suggest that Kelch13 does not mediate artemisinin resistance by modulating proteasome activity but rather by modulating UPR activation and resolution. It was recently reported that Kelch13^mut^ parasites undergo higher levels of autophagy than Kelch13^WT^ under basal conditions (63), which would aid in disposing of damaged proteins, thus complementing any deficiencies in proteasome-mediated protein degradation.

Nevertheless, the proteasome may play a critical role in non-Kelch13-mediated artemisinin response. The third major finding of our study is that parasite susceptibility to DHA, mediated by mutations in the proteasome, correlated with a dysfunction in proteasome-mediated protein degradation. Previous studies showed that upon artemisinin treatment, the artemisinin-sensitive parasites 3D7 and PL2, which harbor Kelch13^WT^, had a twofold increase in ubiquitination, while the artemisinin-resistant PL7 strain that harbors Kelch13^mut^ only accumulated ~1.2-fold increased ubiquitination (57). Note that none of these three strains are isogenic, and there are multiple genetic differences between 3D7, PL2, and PL7, including at known drug resistance modulators such as *P. falciparum* multidrug resistance protein 1, *P. falciparum* multidrug resistance protein 2, and *P. falciparum* chloroquine resistance transporter (57). Corroborating these data, we show that parasites with mutations in the β2 subunit and all tested parasites with mutations in 19S RP proteasome subunits had increased susceptibility to DHA and displayed increased K48-linked ubiquitination. These data show that proteasome subunit mutations and consequently proteasome dysfunction increased parasite susceptibility to DHA. The 19S RP mutants tested selectively affected ring and trophozoite-stage DHA susceptibility. In contrast, β2^C31Y/F^ displayed increased sensitivity at the ring, trophozoite, and asynchronous stages (46). These data could indicate that the 20S CP plays an outsized role in parasite artemisinin response. Perhaps in addition to the 20S-19S complex, the 20S-PA28 complex contributes to resolving artemisinin-mediated protein damage. This is supported by previous findings that 3D7 parasites in which PA28 is knocked out display twofold lower DHA IC_50_ values at the early-ring stage (64).

Although increased ubiquitinated polypeptides were observed in DHA-treated proteasome mutants compared to parental strains, we did not detect any differences in proteolytic activities as measured by cleavage of fluorogenic peptidyl substrates. One reason for this discrepancy is that the fluorogenic substrates can freely diffuse into the 20S CP without processing by the 19S RP, whereas the detection of K48-linked ubiquitinated proteins assesses the ability of the 26S proteasome as a whole to process and degrade proteins. Interestingly, peptidyl substrate cleavage showed that at peak plasma concentrations, OZ439 significantly inhibits the β5 activity of Kelch13^R539T^ and Kelch13^C580Y^ proteasomes but does not inhibit Kelch13^WT^ proteasomes. This could explain why these artemisinin-resistant parasite strains do not exhibit cross-resistance to OZ439 (54, 65). OZ439 also inhibited the β5 catalytic activity of β2^C31F^ significantly more than in the parental Kelch13^C580Y^, which supports our previous data showing that OZ439-treated β2^C31F^ has the greatest decrease in RSA values compared to parental and other 20S CP mutants (46).

**Fig 6:**
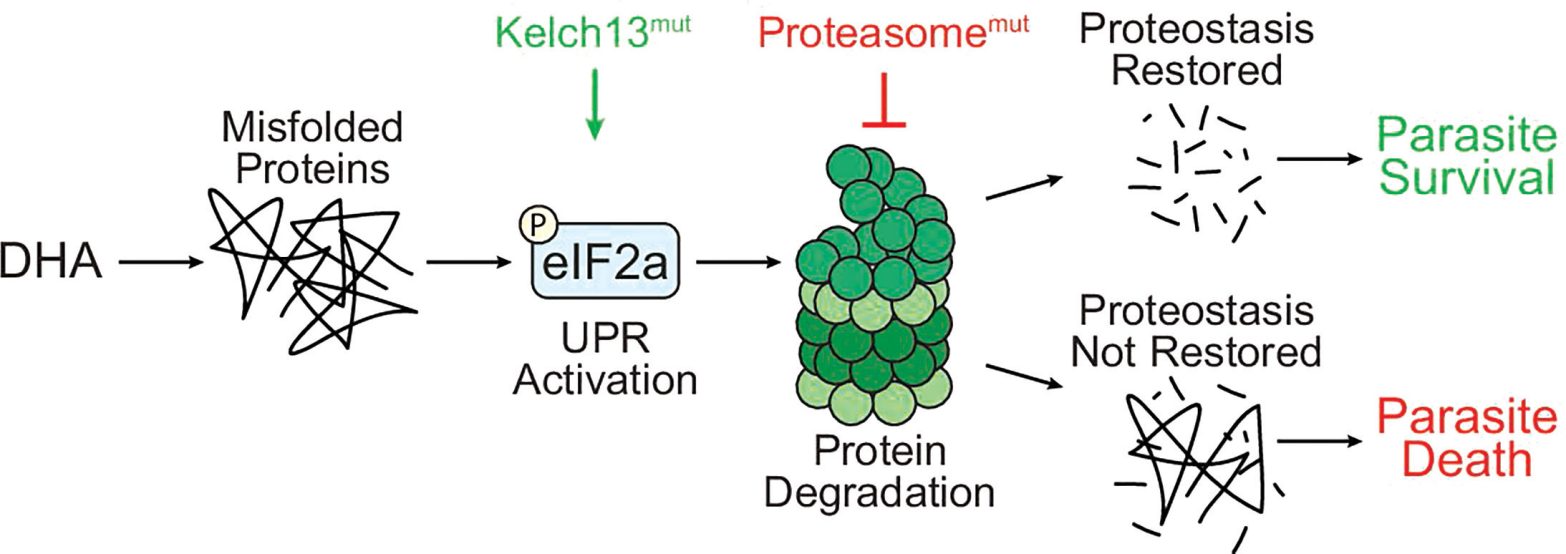
Model for the involvement of parasite UPR and UPS in response to artemisinins. DHA causes an accumulation of misfolded and damaged proteins in the parasite. This triggers activation of the UPR, which allows parasites to temporarily halt protein translation to devote resources to mitigate this damage. Kelch13 regulates the UPR, and Kelch13^mut^ parasites demonstrate more robust and quicker UPR activation at the trophozoite stage, and a quicker UPR resolution following removal of DHA. These signaling processes allow Kelch13^mut^ to activate protective stress responses while timely resuming protein production. The proteasome plays a critical role in parasite recovery by reducing the misfolded protein burden. Mutations in the 19S RP or 20S CP compromise proteasome-mediated protein degradation. Parasites survive DHA treatment if they possess a functional UPR and UPS, which work together to restore proteostasis.

The data presented here indicate that (i) antimalarial compounds that synergize with proteasome inhibitors perturb parasite proteostasis, (ii) early parasite UPR signaling in response to DHA dictates eventual survival outcomes, and (iii) parasite susceptibility to DHA correlates with a dysfunction in proteasome-mediated protein degradation. In summary, DHA-mediated misfolded proteins trigger UPR activation, which is modulated by Kelch13. The proteasome plays a critical role in parasite recovery by reducing the misfolded protein burden, allowing UPR resolution. Parasites survive if proteostasis is restored, which depends on both the UPR and a functioning proteasome (Fig. 6). We show here and previously that chemical inhibition of the proteasome and mutations in the proteasome increase parasite susceptibility to DHA regardless of Kelch13 genotype (46, 49), highlighting the crucial role of the proteasome in *P. falciparum* survival to artemisinin. These data point to the UPR and UPS, two pillars of proteostasis, as pathways that can be targeted to overcome existing artemisinin resistance.

## MATERIALS AND METHODS

### Parasite identity and propagation

*P. falciparum* strains Kelch13^WT^ and Kelch13^C580Y^ were generated by genetically editing the clinical isolate RF967 (Cam3.II Kelch13^R539T^) using zinc finger nucleases (13). Rpt4^E380*^, Rpn6^E266K^, β2^C31Y^, β2^C31F^, β5^A20S^, and Rpt5^G319S^ were obtained from selection studies with the *Plasmodium*-specific proteasome inhibitors WLL and WLW using either Cam3.II K13^WT^ or K13^C580Y^ (49). Parasites were cultured as previously described (46).

### Parasite stage synchronization

For dose-response assays, 0–3_hpi_ rings and 26–30_hpi_ trophozoites were obtained as previously described (46). To obtain a higher protein yield for Western blots and peptidyl substrate cleavage assays, early rings and trophozoites were obtained as described in reference 66. Briefly, cultures were treated with 5% sorbitol a total of three times. Cultures were incubated for 12 h between first and second treatments and then 36 h between second and third treatments. 26–30_hpi_ trophozoites were obtained using two treatments of 5% sorbitol 12 h apart. Following the second treatment, parasites were cultured for an additional 12 h.

### Western blots

Trophozoites were exposed to 50 µM CQ, 2.5 µM WLL, 50 nM DHA, 500 nM OZ439, 5 µM b-AP15, or 500 nM MB, which reflect 5 × IC_50_ values. Rings were exposed to 50 or 700 nM DHA. DMSO concentration for all treatments did not exceed 0.2%. Parasites were released from RBCs using 0.15% saponin (Acros Organics) and then washed three times with 1× PBS at 4°C. Parasites were lysed with 1% Triton X-100 (Thermo Fisher Scientific), 5% glycerol (Thermo Fisher Scientific), 20 mM MgCl_2_ (Sigma-Aldrich, St. Louis, MO, USA), 200 mM KCl (Sigma-Aldrich), and 25 mM HEPES (pH 7.4) on ice for 15 min with intermittent vortexing. Samples were centrifuged at 14,000 × *g* for 10 min at 4°C, and supernatants were transferred to a new microcentrifuge tube. Protein concentration was determined using a Pierce BCA assay (Thermo Fisher Scientific). Samples were flash-frozen with a dry ice and ethanol bath and then stored at −20°C until ready to use. Parasite lysates were mixed with 4× Laemmli SDS sample buffer (Thermo Fisher Scientific) for a final concentration of 1× and boiled at 95°C for 5 min. Equal amounts of parasite proteins, ranging from 2 to 5 µg per lane per blot, were loaded on 4%–20% Criterion TGX Stain-Free gels (Bio-Rad, Hercules, CA, USA) and run at 100 V for 1.5 h, then wet transferred at 10 mA at 4°C for 1.5 h to PVDF membranes (Immobilon-P, Millipore Sigma, Burlington, MA, USA). Blots were blocked in 3% BSA (Thermo Fisher Scientific) in 1× TBS-T and then probed with 1:1,000 dilutions of primary antibodies overnight at 4°C. Blots were washed three times with 1× TBS-T and incubated with secondary HRP-conjugated antibodies at 1:10,000 dilutions for 1 h at room temperature. Primary antibodies were used in the following order: phospho-eIF2α (Cell Signaling Technologies [CST], Danvers, MA, USA, catalog number: 119A11, rabbit]) total eIF2α (CST, catalog number: D7D3, rabbit), K48-linked ubiquitin (CST, catalog number: D9D5, rabbit), and BiP (catalog number: MRA-1247, rat). The following reagents were obtained through BEI Resources, NIAID, NIH: polyclonal anti-*Plasmodium falciparum* PfGRP78 (BiP), anti-SGDEDVDSDEL peptide (antiserum, rat), MRA-1247. Goat anti-rabbit IgG secondary antibody, HRP (catalog number: A16110), goat anti-rat IgG secondary antibody, and HRP (catalog number: 31470) were purchased from Thermo Fisher Scientific. After washing four times with 1× TBS-T, blots were visualized using Immobilon Western Chemiluminescent HRP substrate (Millipore Sigma). Blots were stripped with Restore PLUS Western Blot Stripping Buffer (Thermo Fisher Scientific) between antibodies of the same species. Densitometry was performed with ImageJ version 1.53K. Statistical significance was analyzed with GraphPad version 9 using a two-tailed paired *t*-test.

### Peptidyl substrate cleavage assays

Trophozoites (26–30_hpi_) were treated with DMSO, 50 nM DHA, 700 nM DHA, 300 nM OZ439, 3 µM OZ439, or 2.5 µM WLL for 3 h under hypoxic conditions. DMSO concentration for all treatments did not exceed 0.2%. Parasites were released from RBCs using 0.15% saponin and washed three times with 1× PBS at 4°C. Parasites were lysed with 1× NP-40 lysis buffer (Thermo Fisher Scientific) supplemented with 500 µM MgCl_2_ on ice for 15 min with intermittent vortexing. Samples were centrifuged at 14,000 × *g* for 10 min at 4°C, and supernatants were transferred to a new microcentrifuge tube. Protein concentration was determined using a Pierce BCA assay. Samples were flash-frozen with dry ice and ethanol bath and stored at −20°C until ready to use. OptiPlate-96 black plates (PerkinElmer, Waltham, MA, USA) were placed on ice, and 100 µL assay buffer (50 mM Tris [pH 7.5], 40 mM KCl, 5 mM MgCl_2_, 0.5 mM ATP [TCI America, Portland, OR, USA], 1 mM DTT [Thermo Fisher Scientific], and 0.5% BSA) was added per well. Ten micrograms of parasite lysate was added to assay for β1 activity using 3 µM Ac-Nle-Pro-Nle-Asp-AMC (Ac-nLPnLD-AMC) substrate. Twenty micrograms of lysate was used to assay for β2 activity using 750 nM Ac-Arg-Leu-Arg-AMC (Ac-RLR-AMC) substrate. Ten micrograms of lysate was used to assay β5 activity using 6 µM succinyl-Leu-Leu-Val-Tyr-AMC (Suc-LLVY-AMC) substrate. All fluorogenic peptidyl substrates were purchased from Cayman Chemical (Ann Arbor, MI, USA). PBS (1×) was added to obtain a final volume of 300 µL. Samples were mixed and then read on a TECAN Spark (Morrisville, NC, USA) microplate reader pre-warmed to 37°C at 360/480 excitation/emission (ex/em). Readings were taken every 3 min for 2 h or until fluorescence exceeded the detection maxima. To determine activity, relative fluorescence was plotted over time and the slope of the line was determined in Microsoft Excel. At least three biological replicates were performed for each substrate. Student’s *t*-tests were used to determine differences in relative activity.

### Growth inhibition and ring-stage survival assays

Twofold serial drug dilutions were performed in 96-well plates (Thermo Fisher Scientific), and parasites were seeded at 1% hematocrit and 0.2% parasitemia in 200 µL total per well. For 0–3_hpi_ ring and 26–30_hpi_ trophozoite dose-response assays, parasites were treated for 3 h in U-bottom plates. Three to four washes were performed by centrifuging 96-well plates at 1,500 rpm for 1 min, removing media, and adding 190 µL media per well. Then, the culture was transferred to a new flat-bottom 96-well plate, and plates were incubated for 66 h under normal culturing conditions. For asynchronous dose-response assays, parasites were treated for 72 h in flat-bottom plates. Viable parasites were quantified either by flow cytometry (67) or high-content imaging (68). IC_50_ values were calculated in GraphPad Prism version 9.4.1 using non-linear regression analysis. The percentage of survival for RSAs was calculated by dividing the parasitemia of parasites treated with 700 nM DHA by the parasitemia of mock-treated parasites (13). For 19S RP mutant IC_50_ values, outliers were identified and excluded based on a Grubb’s test with an alpha = 0.2. At least four independent biological replicates were performed for each assay, and statistical significance was examined by Mann-Whitney *U* tests.

### Antimalarials and drug compounds

DHA was purchased from Sigma-Aldrich. OZ439 was kindly provided by Professor Jonathan L. Vennerstrom (University of Nebraska Medical Center). MB and CQ were purchased from Thermo Fisher Scientific. Epoxomicin was purchased from APExBIO (Houston, TX, USA). b-AP15 was purchased from Calbiochem (San Diego, CA, USA). WLL was kindly provided by Professor Mathew Bogyo (Stanford School of Medicine).

## Data Availability

Requests for data or materials should be addressed to the corresponding author.

## SUPPLEMENTAL MATERIAL

Supplemental material msphere.00371-24-s0001.pdf Figures S1-S6 and Table S1.

ASM does not own the copyrights to Supplemental Material that may be linked to, or accessed through, an article. The authors have granted ASM a non-exclusive, world-wide license to publish the Supplemental Material files. Please contact the corresponding author directly for reuse.

## References

[1] WHO. 2023. World malaria report. Available from: https://www.who.int/publications/i/item/9789240086173.283

[2] Asua V, Conrad MD, Aydemir O, Duvalsaint M, Legac J, Duarte E, Tumwebaze P, Chin DM, Cooper RA, Yeka A, Kamya MR, Dorsey G, Nsobya SL, Bailey J, Rosenthal PJ. 2021. Changing prevalence of potential mediators of aminoquinoline, antifolate, and artemisinin resistance across Uganda. J Infect Dis 223:985–994. doi:10.1093/infdis/jiaa687 33146722 PMC8006419

[3] Balikagala B, Fukuda N, Ikeda M, Katuro OT, Tachibana SI, Yamauchi M, Opio W, Emoto S, Anywar DA, Kimura E, Palacpac NMQ, Odongo-Aginya EI, Ogwang M, Horii T, Mita T. 2021. Evidence of artemisinin-resistant malaria in Africa. N Engl J Med 385:1163–1171. doi:10.1056/NEJMoa2101746 34551228

[4] Uwimana A, Umulisa N, Venkatesan M, Svigel SS, Zhou Z, Munyaneza T, Habimana RM, Rucogoza A, Moriarty LF, Sandford R, et al.. 2021. Association of Plasmodium falciparum Kelch13 R561H genotypes with delayed parasite clearance in Rwanda: an open-label, single-arm, multicentre, therapeutic efficacy study. Lancet Infect Dis 21:1120–1128. doi:10.1016/S1473-3099(21)00142-0 33864801 PMC10202849

[5] Tumwebaze PK, Conrad MD, Okitwi M, Orena S, Byaruhanga O, Katairo T, Legac J, Garg S, Giesbrecht D, Smith SR, Ceja FG, Nsobya SL, Bailey JA, Cooper RA, Rosenthal PJ. 2022. Decreased susceptibility of Plasmodium falciparum to both dihydroartemisinin and lumefantrine in northern Uganda. Nat Commun 13:6353. doi:10.1038/s41467-022-33873-x 36289202 PMC9605985

[6] Straimer J, Gandhi P, Renner KC, Schmitt EK. 2022. High prevalence of Plasmodium falciparum K13 mutations in Rwanda Is associated with slow parasite clearance after treatment with artemether-lumefantrine. J Infect Dis 225:1411–1414. doi:10.1093/infdis/jiab352 34216470 PMC9016418

[7] Bakari C, Mandara CI, Madebe RA, Seth MD, Ngasala B, Kamugisha E, Ahmed M, Francis F, Bushukatale S, Chiduo M, et al.. 2024. Trends of Plasmodium falciparum molecular markers associated with resistance to artemisinins and reduced susceptibility to lumefantrine in Mainland Tanzania from 2016 to 2021. Malar J 23:71. doi:10.1186/s12936-024-04896-0 38461239 PMC10924419

[8] Mesia Kahunu G, Wellmann Thomsen S, Wellmann Thomsen L, Muhindo Mavoko H, Mitashi Mulopo P, Filtenborg Hocke E, Mandoko Nkoli P, Baraka V, Minja DTR, Mousa A, Roper C, Mbongi Moke D, Mumba Ngoyi D, Mukomena Sompwe E, Muyembe Tanfum JJ, Hansson H, Alifrangis M. 2024. Identification of the PfK13 mutations R561H and P441L in the democratic republic of Congo. Int J Infect Dis 139:41–49. doi:10.1016/j.ijid.2023.11.026 38016502

[9] van Loon W, Schallenberg E, Igiraneza C, Habarugira F, Mbarushimana D, Nshimiyimana F, Ngarambe C, Ntihumbya JB, Ndoli JM, Mockenhaupt FP. 2024. Escalating Plasmodium falciparum K13 marker prevalence indicative of artemisinin resistance in southern Rwanda. Antimicrob Agents Chemother 68:e0129923. doi:10.1128/aac.01299-23 38092677 PMC10869333

[10] Conrad MD, Asua V, Garg S, Giesbrecht D, Niaré K, Smith S, Namuganga JF, Katairo T, Legac J, Crudale RM, Tumwebaze PK, Nsobya SL, Cooper RA, Kamya MR, Dorsey G, Bailey JA, Rosenthal PJ. 2023. Evolution of partial resistance to artemisinins in malaria parasites in Uganda. N Engl J Med 389:722–732. doi:10.1056/NEJMoa2211803 37611122 PMC10513755

[11] Ariey F, Witkowski B, Amaratunga C, Beghain J, Langlois A-C, Khim N, Kim S, Duru V, Bouchier C, Ma L, et al.. 2014. A molecular marker of artemisinin-resistant Plasmodium falciparum malaria. Nature New Biol 505:50–55. doi:10.1038/nature12876 PMC5007947 24352242

[12] Ashley EA, Dhorda M, Fairhurst RM, Amaratunga C, Lim P, Suon S, Sreng S, Anderson JM, Mao S, Sam B, et al.. 2014. Spread of artemisinin resistance in Plasmodium falciparum malaria. N Engl J Med 371:411–423. doi:10.1056/NEJMoa1314981 25075834 PMC4143591

[13] Straimer J, Gnädig NF, Witkowski B, Amaratunga C, Duru V, Ramadani AP, Dacheux M, Khim N, Zhang L, Lam S, Gregory PD, Urnov FD, Mercereau-Puijalon O, Benoit-Vical F, Fairhurst RM, Ménard D, Fidock DA. 2015. Drug resistance. K13-propeller mutations confer artemisinin resistance in Plasmodium falciparum clinical isolates. Science 347:428–431. doi:10.1126/science.1260867 25502314 PMC4349400

[14] Anderson TJC, Nair S, McDew-White M, Cheeseman IH, Nkhoma S, Bilgic F, McGready R, Ashley E, Pyae Phyo A, White NJ, Nosten F. 2017. Population parameters underlying an ongoing soft sweep in southeast asian malaria parasites. Mol Biol Evol 34:131–144. doi:10.1093/molbev/msw228 28025270 PMC5216669

[15] Zaw MT, Lin Z, Emran NA. 2020. Importance of Kelch 13 C580Y mutation in the studies of artemisinin resistance in Plasmodium falciparum in greater Mekong Subregion. J Microbiol Immunol Infect 53:676–681. doi:10.1016/j.jmii.2019.07.006 31563454

[16] Noedl H, Se Y, Schaecher K, Smith BL, Socheat D, Fukuda MM. 2008. Evidence of artemisinin-resistant malaria in western Cambodia. N Engl J Med 359:2619–2620. doi:10.1056/NEJMc0805011 19064625

[17] Dondorp AM, Nosten F, Yi P, Das D, Phyo AP, Tarning J, Lwin KM, Ariey F, Hanpithakpong W, Lee SJ, Ringwald P, Silamut K, Imwong M, Chotivanich K, Lim P, Herdman T, An SS, Yeung S, Singhasivanon P, Day NPJ, Lindegardh N, Socheat D, White NJ. 2009. Artemisinin resistance in Plasmodium falciparum malaria. N Engl J Med 361:455–467. doi:10.1056/NEJMoa0808859 19641202 PMC3495232

[18] Madamet M, Kounta MB, Wade KA, Lo G, Diawara S, Fall M, Bercion R, Nakoulima A, Fall KB, Benoit N, Gueye MW, Fall B, Diatta B, Pradines B. 2017. Absence of association between polymorphisms in the K13 gene and the presence of Plasmodium falciparum parasites at day 3 after treatment with artemisinin derivatives in Senegal. Int J Antimicrob Agents 49:754–756. doi:10.1016/j.ijantimicag.2017.01.032 28450175

[19] Henrici RC, Schalkwyk DA, Sutherland CJ. 2019. Modification of pfap2mu and pfubp1 markedly reduces ring-stage susceptibility of Plasmodium falciparum to artemisinin in vitro*.* Antimicrob Agents Chemother 64:1. doi:10.1128/AAC.01542-19 PMC7187599 31636063

[20] Demas AR, Sharma AI, Wong W, Early AM, Redmond S, Bopp S, Neafsey DE, Volkman SK, Hartl DL, Wirth DF. 2018. Mutations in Plasmodium falciparum actin-binding protein coronin confer reduced artemisinin susceptibility. Proc Natl Acad Sci U S A 115:12799–12804. doi:10.1073/pnas.1812317115 30420498 PMC6294886

[21] Das S, Kar A, Manna S, Mandal S, Mandal S, Das S, Saha B, Hati AK. 2021. Artemisinin combination therapy fails even in the absence of Plasmodium falciparum Kelch13 gene polymorphism in central India. Sci Rep 11:9946. doi:10.1038/s41598-021-89295-0 33976269 PMC8113598

[22] Kaiser M, Wittlin S, Nehrbass-Stuedli A, Dong Y, Wang X, Hemphill A, Matile H, Brun R, Vennerstrom JL. 2007. Peroxide bond-dependent antiplasmodial specificity of artemisinin and OZ277 (RBx11160). Antimicrob Agents Chemother 51:2991–2993. doi:10.1128/AAC.00225-07 17562801 PMC1932508

[23] Xie SC, Dogovski C, Hanssen E, Chiu F, Yang T, Crespo MP, Stafford C, Batinovic S, Teguh S, Charman S, Klonis N, Tilley L. 2016. Haemoglobin degradation underpins the sensitivity of early ring stage Plasmodium falciparum to artemisinins. J Cell Sci 129:406–416. doi:10.1242/jcs.178830 26675237 PMC4732288

[24] Rosenthal MR, Ng CL. 2020. Plasmodium falciparum artemisinin resistance: the effect of heme, protein damage, and parasite cell stress response. ACS Infect Dis 6:1599–1614. doi:10.1021/acsinfecdis.9b00527 32324369 PMC8528195

[25] Birnbaum J, Flemming S, Reichard N, Soares AB, Mesén-Ramírez P, Jonscher E, Bergmann B, Spielmann T. 2017. A genetic system to study Plasmodium falciparum protein function. Nat Methods 14:450–456. doi:10.1038/nmeth.4223 28288121

[26] Birnbaum J, Scharf S, Schmidt S, Jonscher E, Hoeijmakers WAM, Flemming S, Toenhake CG, Schmitt M, Sabitzki R, Bergmann B, Fröhlke U, Mesén-Ramírez P, Blancke Soares A, Herrmann H, Bártfai R, Spielmann T. 2020. A Kelch13-defined endocytosis pathway mediates artemisinin resistance in malaria parasites. Science 367:51–59. doi:10.1126/science.aax4735 31896710

[27] Yang T, Yeoh LM, Tutor MV, Dixon MW, McMillan PJ, Xie SC, Bridgford JL, Gillett DL, Duffy MF, Ralph SA, McConville MJ, Tilley L, Cobbold SA. 2019. Decreased K13 abundance reduces hemoglobin catabolism and proteotoxic stress, underpinning artemisinin resistance. Cell Rep 29:2917–2928. doi:10.1016/j.celrep.2019.10.095 31775055

[28] Heller LE, Goggins E, Roepe PD. 2018. Dihydroartemisinin-ferriprotoporphyrin IX adduct abundance in Plasmodium falciparum malarial parasites and the relationship to emerging artemisinin resistance. Biochemistry 57:6935–6945. doi:10.1021/acs.biochem.8b00960 30512926

[29] Heller LE, Roepe PD. 2018. Quantification of free ferriprotoporphyrin IX heme and hemozoin for artemisinin sensitive versus delayed clearance phenotype Plasmodium falciparum malarial parasites. Biochemistry 57:6927–6934. doi:10.1021/acs.biochem.8b00959 30513202

[30] Wang J, Zhang C-J, Chia WN, Loh CCY, Li Z, Lee YM, He Y, Yuan L-X, Lim TK, Liu M, Liew CX, Lee YQ, Zhang J, Lu N, Lim CT, Hua Z-C, Liu B, Shen H-M, Tan KSW, Lin Q. 2015. Haem-activated promiscuous targeting of artemisinin in Plasmodium falciparum. Nat Commun 6:10111. doi:10.1038/ncomms10111 26694030 PMC4703832

[31] Ismail HM, Barton VE, Panchana M, Charoensutthivarakul S, Biagini GA, Ward SA, O’Neill PM. 2016. A click chemistry-based Proteomic approach reveals that 1,2,4-trioxolane and artemisinin antimalarials share a common protein alkylation profile. Angew Chem Int Ed Engl 55:6401–6405. doi:10.1002/anie.201512062 27089538 PMC4934138

[32] Jourdan J, Walz A, Matile H, Schmidt A, Wu J, Wang X, Dong Y, Vennerstrom JL, Schmidt RS, Wittlin S, Mäser P. 2019. Stochastic protein alkylation by antimalarial peroxides. ACS Infect Dis 5:2067–2075. doi:10.1021/acsinfecdis.9b00264 31626733

[33] Bridgford JL, Xie SC, Cobbold SA, Pasaje CFA, Herrmann S, Yang T, Gillett DL, Dick LR, Ralph SA, Dogovski C, Spillman NJ, Tilley L. 2018. Artemisinin kills malaria parasites by damaging proteins and inhibiting the proteasome. Nat Commun 9:3801. doi:10.1038/s41467-018-06221-1 30228310 PMC6143634

[34] Chen MZ, Moily NS, Bridgford JL, Wood RJ, Radwan M, Smith TA, Song Z, Tang BZ, Tilley L, Xu X, Reid GE, Pouladi MA, Hong Y, Hatters DM. 2017. A thiol probe for measuring unfolded protein load and proteostasis in cells. Nat Commun 8:474. doi:10.1038/s41467-017-00203-5 28883394 PMC5589734

[35] Zhang M, Mishra S, Sakthivel R, Rojas M, Ranjan R, Sullivan WJ Jr, Fontoura BMA, Ménard R, Dever TE, Nussenzweig V. 2012. PK4, a eukaryotic initiation factor 2α(eIF2α) kinase, is essential for the development of the erythrocytic cycle of Plasmodium*.* Proc Natl Acad Sci U S A 109:3956–3961. doi:10.1073/pnas.1121567109 22355110 PMC3309761

[36] Zhang M, Gallego-Delgado J, Fernandez-Arias C, Waters NC, Rodriguez A, Tsuji M, Wek RC, Nussenzweig V, Sullivan WJ Jr. 2017. Inhibiting the Plasmodium eIF2α kinase PK4 prevents artemisinin-induced latency. Cell Host Microbe 22:766–776. doi:10.1016/j.chom.2017.11.005 29241041 PMC5869688

[37] Hetz C, Zhang K, Kaufman RJ. 2020. Mechanisms, regulation and functions of the unfolded protein response. Nat Rev Mol Cell Biol 21:421–438. doi:10.1038/s41580-020-0250-z 32457508 PMC8867924

[38] Gosline SJC, Nascimento M, McCall L-I, Zilberstein D, Thomas DY, Matlashewski G, Hallett M. 2011. Intracellular eukaryotic parasites have a distinct unfolded protein response. PLoS One 6:e19118. doi:10.1371/journal.pone.0019118 21559456 PMC3084755

[39] Wang L, Delahunty C, Fritz-Wolf K, Rahlfs S, Helena Prieto J, Yates JR, Becker K. 2015. Characterization of the 26S proteasome network in Plasmodium falciparum. Sci Rep 5:17818. doi:10.1038/srep17818 26639022 PMC4671066

[40] Dikic I. 2017. Proteasomal and autophagic degradation systems. Annu Rev Biochem 86:193–224. doi:10.1146/annurev-biochem-061516-044908 28460188

[41] Finley D, Chen X, Walters KJ. 2016. Gates, channels, and switches: elements of the proteasome machine. Trends Biochem Sci 41:77–93. doi:10.1016/j.tibs.2015.10.009 26643069 PMC4706478

[42] Li H, O’Donoghue AJ, van der Linden WA, Xie SC, Yoo E, Foe IT, Tilley L, Craik CS, da Fonseca PCA, Bogyo M. 2016. Structure- and function-based design of Plasmodium-selective proteasome inhibitors. Nature New Biol 530:233–236. doi:10.1038/nature16936 PMC4755332 26863983

[43] Xie SC, Dick LR, Gould A, Brand S, Tilley L. 2019. The proteasome as a target for protozoan parasites. Expert Opin Ther Targets 23:903–914. doi:10.1080/14728222.2019.1685981 31679410

[44] Gantt SM, Myung JM, Briones MR, Li WD, Corey EJ, Omura S, Nussenzweig V, Sinnis P. 1998. Proteasome inhibitors block development of Plasmodium spp. Antimicrob Agents Chemother 42:2731–2738. doi:10.1128/AAC.42.10.2731 9756786 PMC105928

[45] Czesny B, Goshu S, Cook JL, Williamson KC. 2009. The proteasome inhibitor epoxomicin has potent Plasmodium falciparum gametocytocidal activity. Antimicrob Agents Chemother 53:4080–4085. doi:10.1128/AAC.00088-09 19651911 PMC2764187

[46] Rosenthal MR, Ng CL. 2021. A proteasome mutation sensitizes P. falciparum Cam3.II K13 C580Y parasites to DHA and OZ439. ACS Infect Dis 7:1923–1931. doi:10.1021/acsinfecdis.0c00900 33971094 PMC8500539

[47] Mok S, Ashley EA, Ferreira PE, Zhu L, Lin Z, Yeo T, Chotivanich K, Imwong M, Pukrittayakamee S, Dhorda M, et al.. 2015. Drug resistance. Population transcriptomics of human malaria parasites reveals the mechanism of artemisinin resistance. Science 347:431–435. doi:10.1126/science.1260403 25502316 PMC5642863

[48] Mok S, Stokes BH, Gnädig NF, Ross LS, Yeo T, Amaratunga C, Allman E, Solyakov L, Bottrill AR, Tripathi J, Fairhurst RM, Llinás M, Bozdech Z, Tobin AB, Fidock DA. 2021. Artemisinin-resistant K13 mutations rewire Plasmodium falciparum’s intra-erythrocytic metabolic program to enhance survival. Nat Commun 12:530. doi:10.1038/s41467-020-20805-w 33483501 PMC7822823

[49] Stokes BH, Yoo E, Murithi JM, Luth MR, Afanasyev P, da Fonseca PCA, Winzeler EA, Ng CL, Bogyo M, Fidock DA. 2019. Covalent Plasmodium falciparum-selective proteasome inhibitors exhibit a low propensity for generating resistance in vitro and synergize with multiple antimalarial agents. PLoS Pathog 15:e1007722. doi:10.1371/journal.ppat.1007722 31170268 PMC6553790

[50] Finley D. 2009. Recognition and processing of ubiquitin-protein conjugates by the proteasome. Annu Rev Biochem 78:477–513. doi:10.1146/annurev.biochem.78.081507.101607 19489727 PMC3431160

[51] Friedlander R, Jarosch E, Urban J, Volkwein C, Sommer T. 2000. A regulatory link between ER-associated protein degradation and the unfolded-protein response. Nat Cell Biol 2:379–384. doi:10.1038/35017001 10878801

[52] Travers KJ, Patil CK, Wodicka L, Lockhart DJ, Weissman JS, Walter P. 2000. Functional and genomic analyses reveal an essential coordination between the unfolded protein response and ER-associated degradation. Cell 101:249–258. doi:10.1016/s0092-8674(00)80835-1 10847680

[53] Witkowski B, Amaratunga C, Khim N, Sreng S, Chim P, Kim S, Lim P, Mao S, Sopha C, Sam B, Anderson JM, Duong S, Chuor CM, Taylor WRJ, Suon S, Mercereau-Puijalon O, Fairhurst RM, Menard D. 2013. Novel phenotypic assays for the detection of artemisinin-resistant Plasmodium falciparum malaria in Cambodia: in-vitro and ex-vivo drug-response studies. Lancet Infect Dis 13:1043–1049. doi:10.1016/S1473-3099(13)70252-4 24035558 PMC5015432

[54] Straimer J, Gnädig NF, Stokes BH, Ehrenberger M, Crane AA, Fidock DA. 2017. Plasmodium falciparum K13 mutations differentially impact ozonide susceptibility and parasite fitness in vitro. MBio 8:e00172-17. doi:10.1128/mBio.00172-17 28400526 PMC5388803

[55] Ponts N, Saraf A, Chung D-WD, Harris A, Prudhomme J, Washburn MP, Florens L, Le Roch KG. 2011. Unraveling the ubiquitome of the human malaria parasite. J Biol Chem 286:40320–40330. doi:10.1074/jbc.M111.238790 21930698 PMC3220526

[56] Bozdech Z, Llinás M, Pulliam BL, Wong ED, Zhu J, DeRisi JL. 2003. The transcriptome of the intraerythrocytic developmental cycle of Plasmodium falciparum*.* PLoS Biol 1:E5. doi:10.1371/journal.pbio.0000005 12929205 PMC176545

[57] Dogovski C, Xie SC, Burgio G, Bridgford J, Mok S, McCaw JM, Chotivanich K, Kenny S, Gnädig N, Straimer J, Bozdech Z, Fidock DA, Simpson JA, Dondorp AM, Foote S, Klonis N, Tilley L. 2015. Targeting the cell stress response of Plasmodium falciparum to overcome artemisinin resistance. PLoS Biol 13:e1002132. doi:10.1371/journal.pbio.1002132 25901609 PMC4406523

[58] Kisselev AF, Goldberg AL. 2005. Monitoring activity and inhibition of 26S proteasomes with fluorogenic peptide substrates. Methods Enzymol 398:364–378. doi:10.1016/S0076-6879(05)98030-0 16275343

[59] Phyo AP, Jittamala P, Nosten FH, Pukrittayakamee S, Imwong M, White NJ, Duparc S, Macintyre F, Baker M, Möhrle JJ. 2016. Antimalarial activity of artefenomel (OZ439), a novel synthetic antimalarial endoperoxide, in patients with Plasmodium falciparum and Plasmodium vivax malaria: an open-label phase 2 trial. Lancet Infect Dis 16:61–69. doi:10.1016/S1473-3099(15)00320-5 26448141 PMC4700386

[60] Deni I, Stokes BH, Ward KE, Fairhurst KJ, Pasaje CFA, Yeo T, Akbar S, Park H, Muir R, Bick DS, et al.. 2023. Mitigating the risk of antimalarial resistance via covalent dual-subunit inhibition of the Plasmodium proteasome. Cell Chem Biol 30:470–485. doi:10.1016/j.chembiol.2023.03.002 36963402 PMC10198959

[61] Garg S, Kreutzfeld O, Chelebieva S, Tumwebaze PK, Byaruhanga O, Okitwi M, Orena S, Katairo T, Nsobya SL, Conrad MD, Aydemir O, Legac J, Gould AE, Bayles BR, Bailey JA, Duffey M, Lin G, Kirkman LA, Cooper RA, Rosenthal PJ. 2022. Susceptibilities of Ugandan Plasmodium falciparum isolates to proteasome inhibitors. Antimicrob Agents Chemother 66:e0081722. doi:10.1128/aac.00817-22 36094216 PMC9578402

[62] Klonis N, Xie SC, McCaw JM, Crespo-Ortiz MP, Zaloumis SG, Simpson JA, Tilley L. 2013. Altered temporal response of malaria parasites determines differential sensitivity to artemisinin. Proc Natl Acad Sci U S A 110:5157–5162. doi:10.1073/pnas.1217452110 23431146 PMC3612604

[63] Ray A, Mathur M, Choubey D, Karmodiya K, Surolia N. 2022. Autophagy underlies the proteostasis mechanisms of artemisinin resistance in P. falciparum malaria. MBio 13:e0063022. doi:10.1128/mbio.00630-22 35420484 PMC9239046

[64] Xie SC, Metcalfe RD, Hanssen E, Yang T, Gillett DL, Leis AP, Morton CJ, Kuiper MJ, Parker MW, Spillman NJ, Wong W, Tsu C, Dick LR, Griffin MDW, Tilley L. 2019. The structure of the PA28-20S proteasome complex from Plasmodium falciparum and implications for proteostasis. Nat Microbiol 4:1990–2000. doi:10.1038/s41564-019-0524-4 31384003

[65] Baumgärtner F, Jourdan J, Scheurer C, Blasco B, Campo B, Mäser P, Wittlin S. 2017. In vitro activity of anti-malarial ozonides against an artemisinin-resistant isolate. Malar J 16:45. doi:10.1186/s12936-017-1696-0 28122617 PMC5267415

[66] Kite WA, Melendez-Muniz VA, Moraes Barros RR, Wellems TE, Sá JM. 2016. Alternative methods for the Plasmodium falciparum artemisinin ring-stage survival assay with increased simplicity and parasite stage-specificity. Malar J 15:94. doi:10.1186/s12936-016-1148-2 26888201 PMC4756417

[67] Ng CL, Siciliano G, Lee MCS, de Almeida MJ, Corey VC, Bopp SE, Bertuccini L, Wittlin S, Kasdin RG, Le Bihan A, Clozel M, Winzeler EA, Alano P, Fidock DA. 2016. CRISPR-Cas9-modified pfmdr1 protects Plasmodium falciparum asexual blood stages and gametocytes against a class of piperazine-containing compounds but potentiates artemisinin-based combination therapy partner drugs. Mol Microbiol 101:381–393. doi:10.1111/mmi.13397 27073104 PMC4958522

[68] Rosenthal MR, Ng CL. 2023. High-content imaging as a tool to quantify and characterize malaria parasites. Cell Rep Methods 3:100516. doi:10.1016/j.crmeth.2023.100516 37533635 PMC10391350

