# Supplementary material for: Enhanced cell stress response and protein degradation capacity underlie artemisinin resistance in Plasmodium falciparum
Source: mSphere. 2024 Oct 22;9(11):e00371-24. doi: 10.1128/msphere.00371-24 (PMC11580438; doi:10.1128/msphere.00371-24)
Supplement: Supplemental material — Figures S1-S6 and Table S1. [file msphere.00371-24-s0001.pdf]

Enhanced cell stress response and protein degradation capacity underlie artemisinin resistance in *Plasmodium falciparum*

Supplemental Information

Figure S1

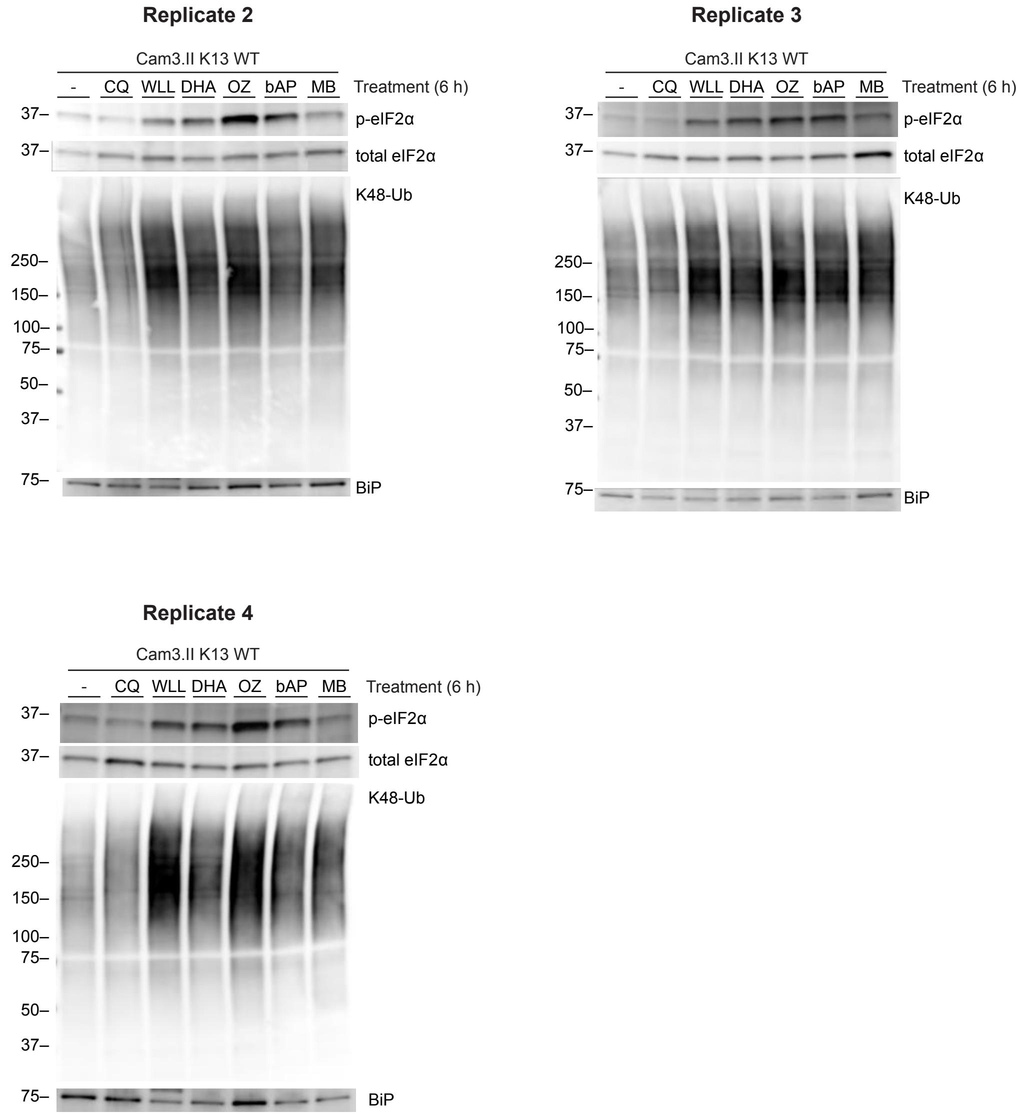

Figure S1. Antimalarial compounds synergistic with proteasome inhibitors disrupt proteostasis. Cam3.II K13^{WT} parasites were synchronized to the trophozoite stage (26-30 hpi) and treated with DMSO, 50 µM chloroquine (CQ), 2.5 µM WLL, 50 nM dihydroartemisinin (DHA), 500 nM OZ439, 5 µM b-AP15, or 500 nM methylene blue (MB). All treatments were at 5×IC_{50} concentrations. Lysates were subject to Western blot and immunoblotted with antibodies against p-eIF2α, total eIF2α, K48-linked ubiquitin, and BiP. Shown are biological replicates relating to Fig. 1.

Figure S2

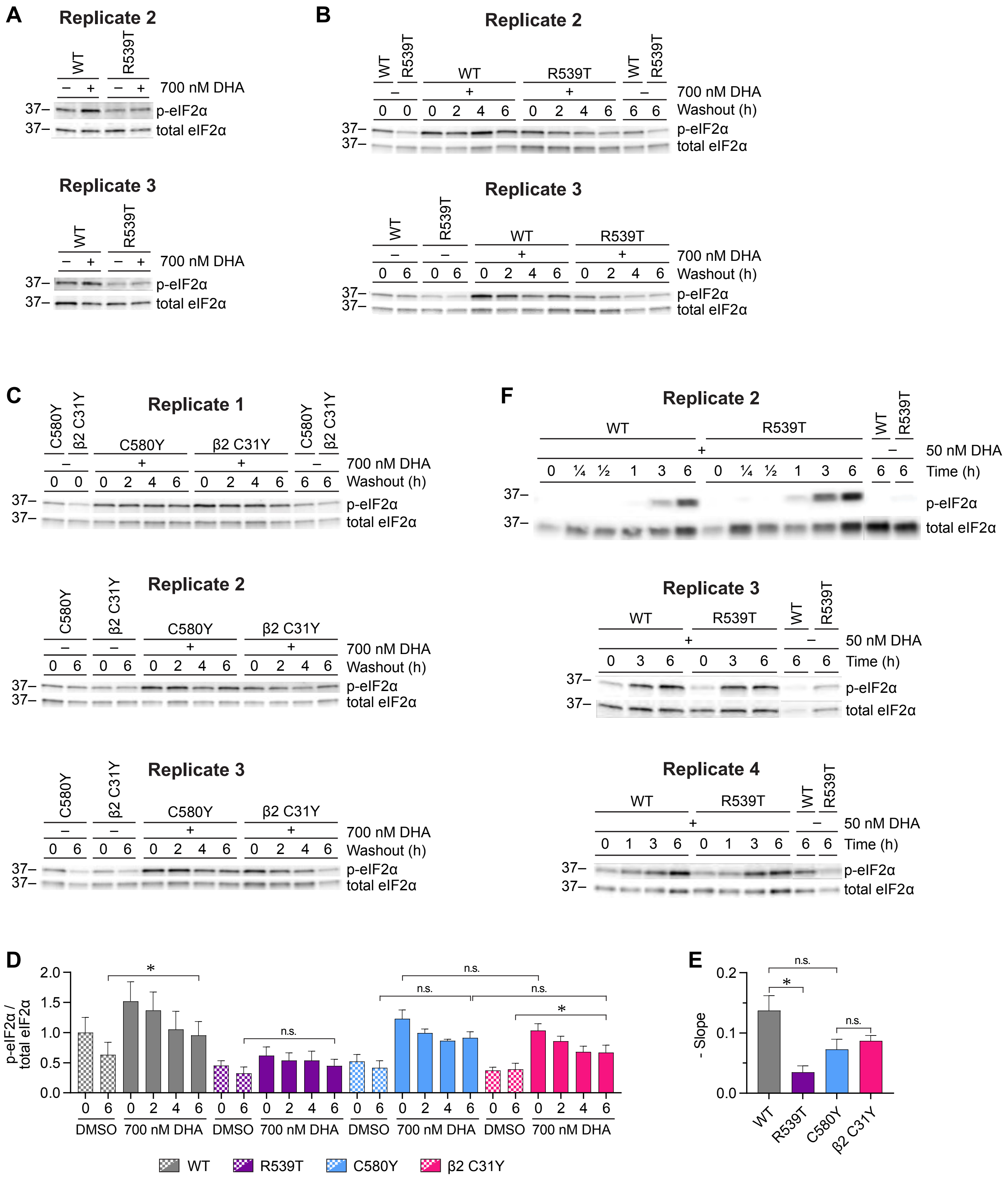

(Legend on next page)

Figure S2. Kelch13^{WT} and Kelch13^{mut} parasites differentially regulate the UPR. (**A**) WT and R539T parasites were synchronized to 0-3 hpi rings and treated with DMSO or 700 nM DHA for 3 h, then lysates were subject to Western blot and immunoblotted with antibodies against p-eIF2α and eIF2α. Shown are biological replicates relating to Fig. 2B and 2C. (**B**) WT and R539T parasites were synchronized to 0-3 hpi rings and treated with DMSO or 700 nM DHA for 3 h. Then drug was washed off and parasites were harvested at the indicated times to monitor UPR resolution. Western blot was performed as described in (A). Shown are biological replicates relating to Fig. 2D-F. (**C**) C580Y and β2^{C31Y} parasites were synchronized to 0-3 hpi rings and treated with DMSO or 700 nM DHA for 3 h. Then drug was washed off and parasites were harvested at the indicated times to monitor UPR resolution. Western blot was performed as described in (A). Shown are all three biological replicates. (**D**) Densitometry analyses was performed using Image J to assess UPR activation by normalizing p-eIF2α to total eIF2α. Bar graphs indicate mean normalized integrated density ± S.E.M. A two-tailed paired *t*-test was used to compare 6 h untreated and DHA-treated parasites. (**E**) Rate of de-phosphorylation of eIF2α following drug removal was calculated over time and the mean negative slope ± S.E.M. was plotted. *$p < 0.05$; n.s. = not significant. (**F**) WT and R539T parasites were synchronized to 26-30 hpi trophozoites, then treated with DMSO or 50 nM DHA for the indicated times. Western blot was performed as described in (A). Shown are biological replicates relating to Fig. 2G and 2H.

Figure S3

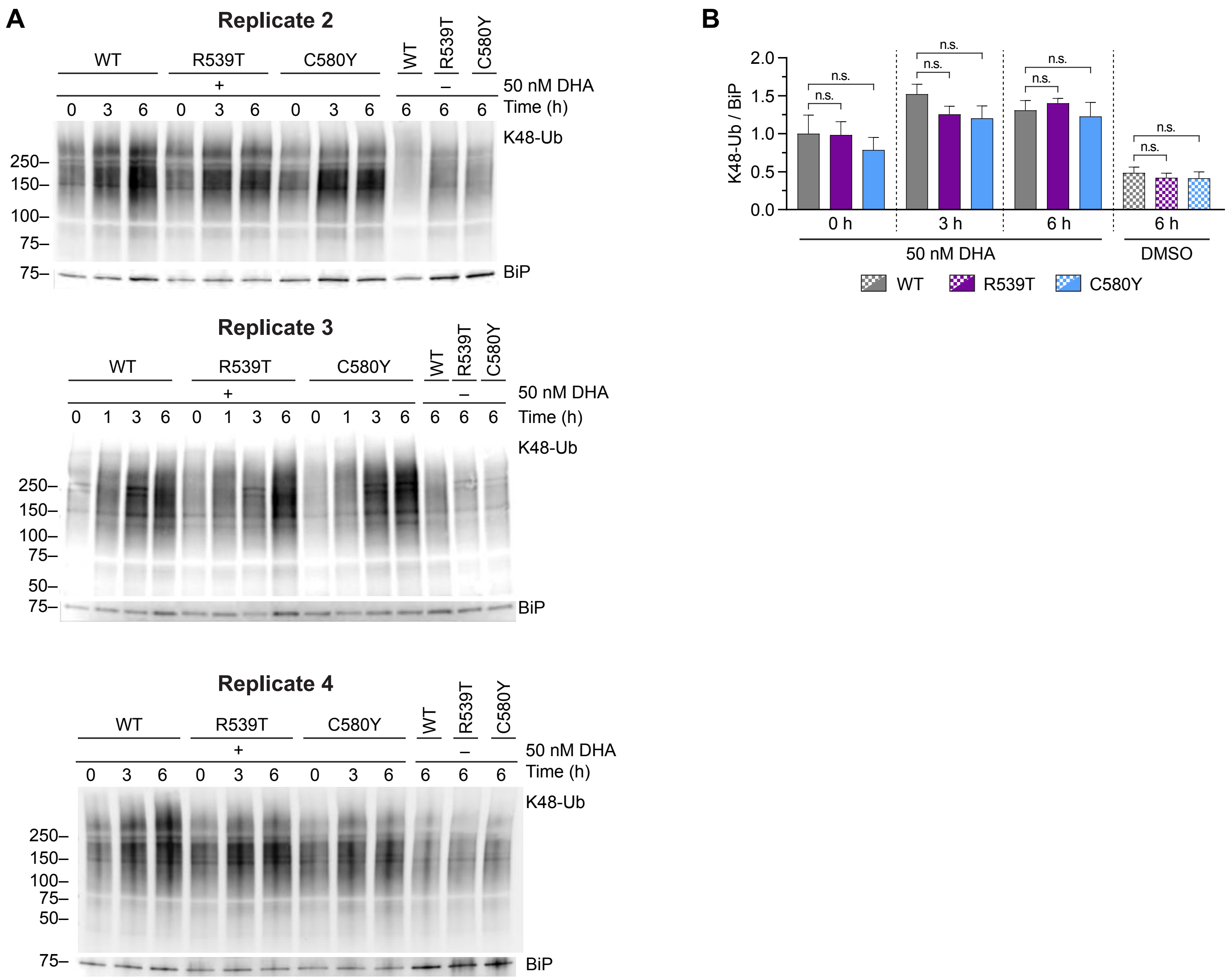

Figure S3. Peroxides DHA and OZ439 inhibit parasite proteasome activity. (**A**) WT, R539T, and C580Y parasites were treated with DMSO or 50 nM DHA for the indicated times. Lysates were subjected to Western blot and immunoblotted with antibodies against K48-linked ubiquitin and BiP. Shown are biological replicates relating to Fig. 3g and 3h. (**B**) Densitometry analyses was performed with Image J and levels of K48-linked ubiquitination was normalized to the loading control BiP. Bar graphs indicate mean normalized integrated density ± S.E.M. A two-tailed paired *t*-test was performed comparing Kelch13^{WT} to Kelch13^{mut} parasites. n.s. = not significant.

Figure S4

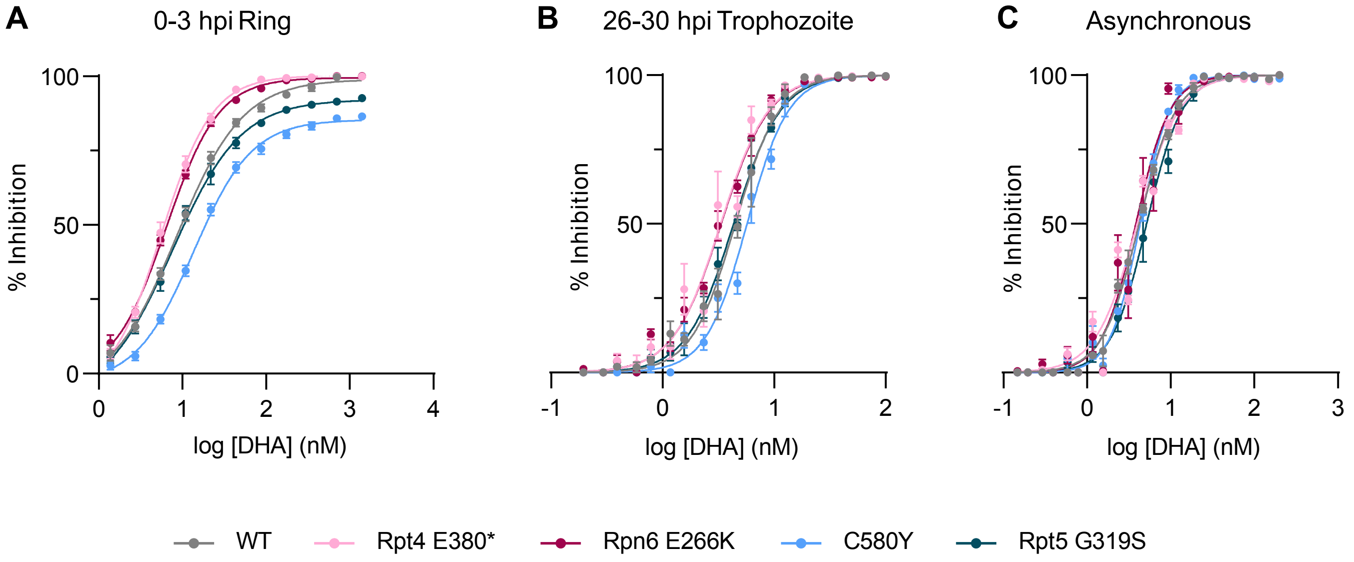

Figure S4. 19S proteasome mutants display increased susceptibility to DHA. (**A**) WT, Rpt4, Rpn6, C580Y, and Rpt5 parasites were tightly synchronized to 0-3 hpi rings, then exposed to a range of DHA concentrations starting at 1400 nM DHA for 3 h. DHA was washed off, and parasitemia was assessed 66 h later. Six independent biological replicates. Data related to Fig. 4A-C. **(B**) 26-30 hpi trophozoites derived from indicated parasites were exposed to a range of DHA concentrations for 3 h, then DHA was washed off and parasitemia assessed 66 h later. Seven independent biological replicates. Data related to Fig. 4D. (**C**) 72 h dose response assays with DHA was performed on asynchronous parasites. Five independent biological replicates. Data related to Fig. 4E. Dose-response curves are shown. Each data point represents mean % inhibition ± S.E.M.

Figure S5

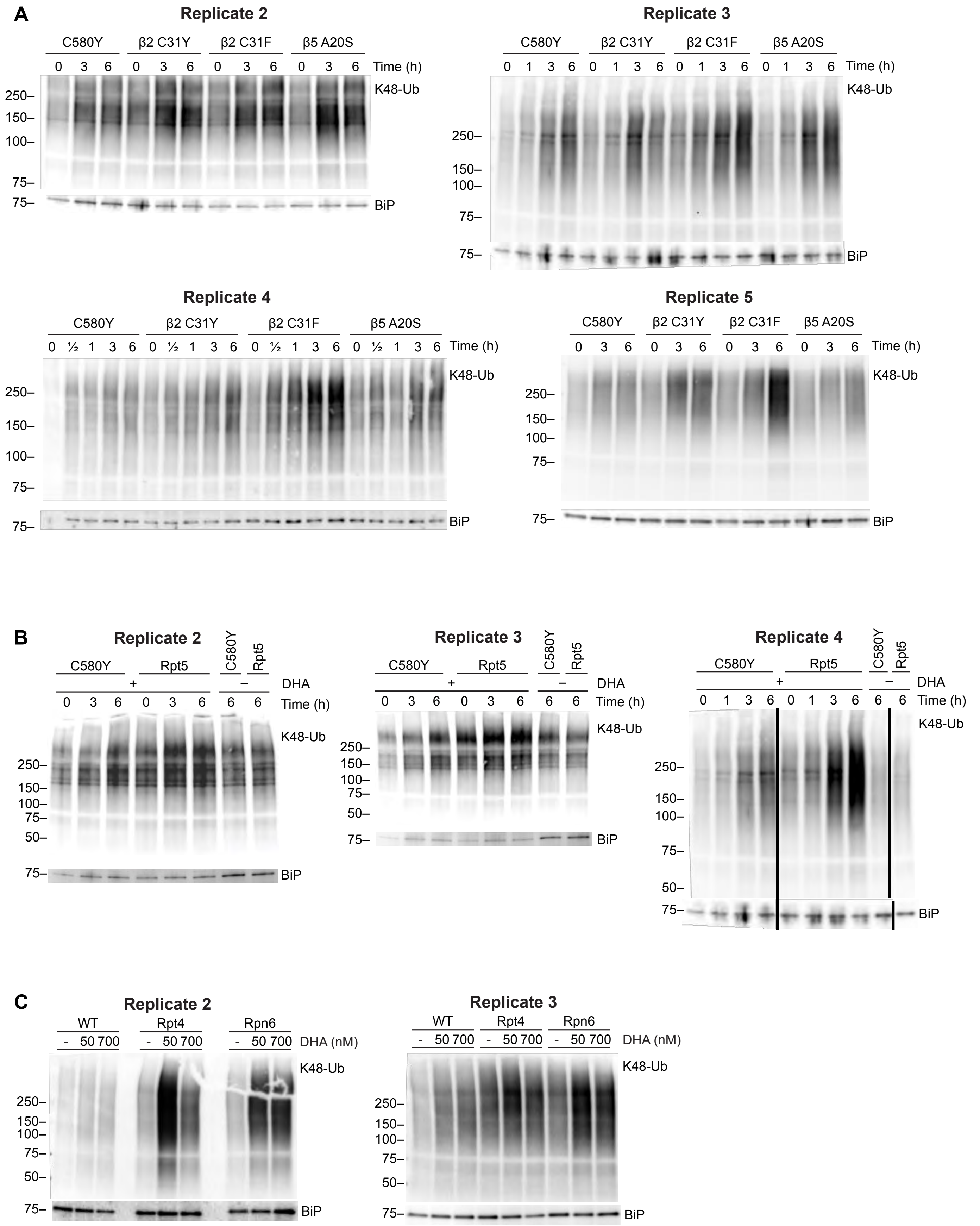

(Legend on next page)

Figure S5. Proteolytic activity of proteasome mutants. (**A**) Trophozoites from C580Y, $\beta2^{C31Y}$, $\beta2^{C31F}$, and $\beta5^{A20S}$ parasites were treated with 50 nM DHA for 0, 3, and 6 h. Lysates were subjected to Western blot and immunoblotted with antibodies against K48-linked ubiquitin and BiP. Shown are biological replicates relating to Fig. 5G and 5H. (**B**) 26-30 hpi trophozoites derived from C580Y and Rpt5 parasites were treated with 50 nM DHA for 0, 3, and 6 h. Western blot performed as described in (A). Shown are biological replicates relating to Fig. 5I and 5J. (**C**) WT, Rpt4, and Rpn6 trophozoites were treated with DMSO, 50 nM, or 700 nM DHA for 3 h. Western blot performed as described in (A). Shown are biological replicates relating to Fig. 5K and 5L.

Figure S6

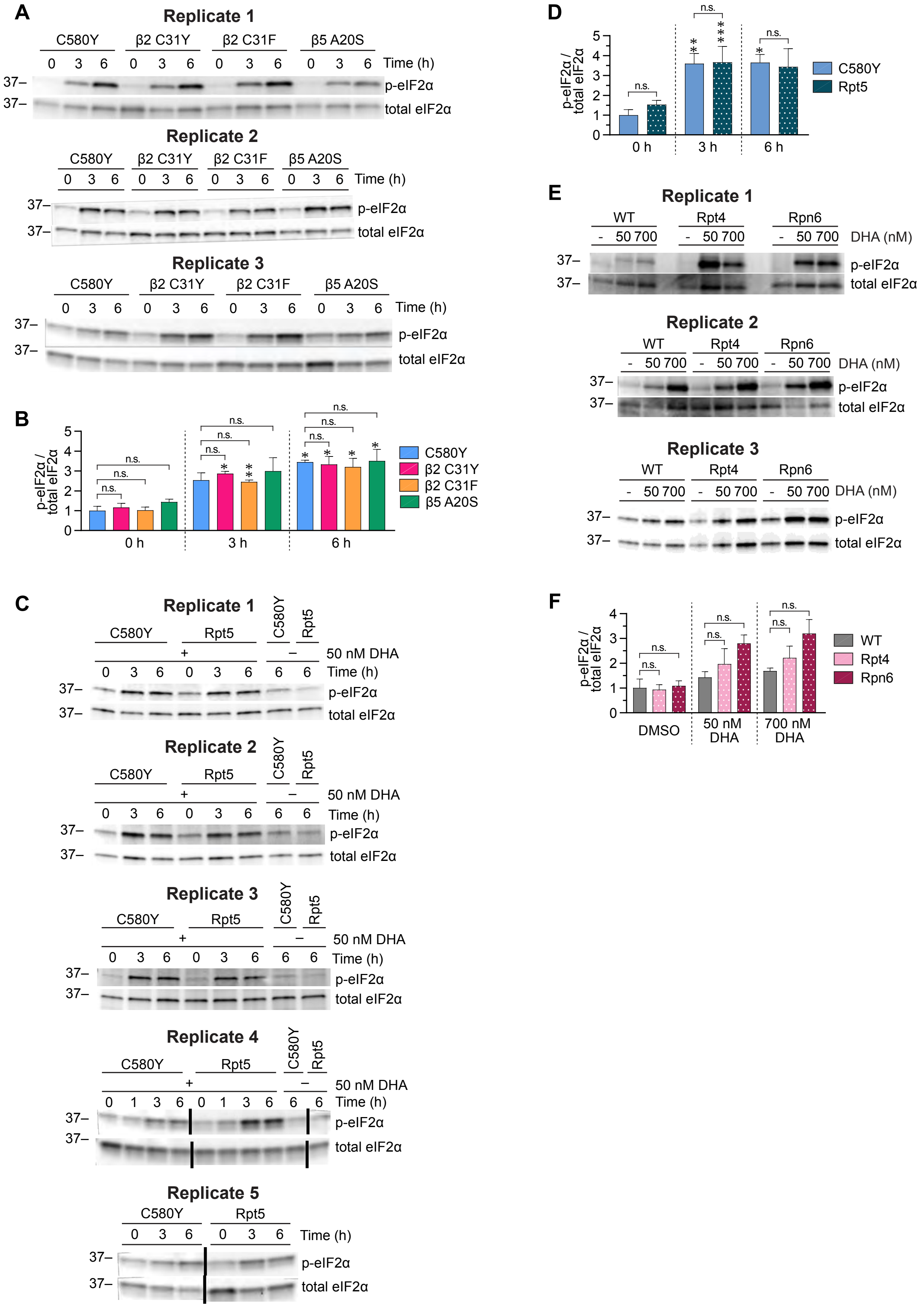

(Legend on next page)

Figure S6. Parental C580Y strain and proteasome mutants do not differentially regulate the UPR. (**A**) C580Y, β2^{C31Y}, β2^{C31F}, and β5^{A20S} parasites were synchronized to 26-30 hpi trophozoites and treated with DMSO or 50 nM DHA for 3 h, then lysates were subject to Western blot and immunoblotted with antibodies against p-eIF2α and eIF2α. All three biological replicates are shown. (**B**) Densitometry analyses was performed using Image J to assess UPR activation by normalizing p-eIF2α to total eIF2α. Bar graphs indicate mean normalized integrated density ± S.E.M. A two-tailed paired *t*-test was used to compare untreated to treated counterparts, indicated above the bar with vertical asterisks. A two-tailed paired *t*-test was also used to compare C580Y parental strain and proteasome mutants, indicated by brackets. (**C**) 26-30 hpi trophozoites derived from C580Y and Rpt5 parasites were treated with DMSO or 50 nM DHA for the indicated times. Western blots were performed as described in (A). All five biological replicates are shown. (**D**) Densitometry analyses and quantitation performed as in (B). (**E**) WT, Rpt4, and Rpn6 parasites were synchronized to 26-30 hpi trophozoites and treated with DMSO, 50 nM, or 700 nM DHA for 3 h, then lysates were subject to Western blot and immunoblotted with antibodies against p-eIF2α and eIF2α. All three biological replicates are shown. (**F**) Densitometry analyses and quantitation performed as in (b). *$p < 0.05$; **$p < 0.01$; ***$p < 0.001$; n.s. = not significant.

Parasite Strain	Previous name (if different)	Method of obtaining parasites	Citation
Cam3.II Kelch13^{WT}	Cam3.II^{rev}	Zinc Finger Nuclease-mediated editing	Straimer, J. *et al*. 2015. K13-propeller mutations confer artemisinin resistance in *Plasmodium falciparum* clinical isolates. Science 347:428-31. PMID: 25502314.
	Cam3.II K13^{WT}	As from Straimer, J. *et al.* 2015	Stokes, B.H. *et al*. 2019. Covalent *Plasmodium falciparum*-selective proteasome inhibitors exhibit a low propensity for generating resistance in vitro and synergize with multiple antimalarial agents. PLoS Pathog 15:e1007722. PMID: 31170268.
Cam3.II Kelch13^{WT} Rpt4^{E380*}	Cam3.II K13^{WT} RPT4^{E380*}	In vitro selection with WLW	Stokes, B.H. *et al*. 2019. Covalent *Plasmodium falciparum*-selective proteasome inhibitors exhibit a low propensity for generating resistance in vitro and synergize with multiple antimalarial agents. PLoS Pathog 15:e1007722. PMID: 31170268.
Cam3.II Kelch13^{WT} Rpn6^{E266K}	Cam3.II K13^{WT} RPN6^{E266K}	In vitro selection with WLW	Stokes, B.H. *et al*. 2019. Covalent *Plasmodium falciparum*-selective proteasome inhibitors exhibit a low propensity for generating resistance in vitro and synergize with multiple antimalarial agents. PLoS Pathog 15:e1007722. PMID: 31170268.
Cam3.II Kelch13^{R539T}	Cam3.II^{R539T}	Original RF 967 parasite isolate	Straimer, J. *et al*. 2015. K13-propeller mutations confer artemisinin resistance in *Plasmodium falciparum* clinical isolates. Science 347:428-31. PMID: 25502314.
	Cam3.II K13^{R539T}	As from Straimer, J. *et al.* 2015	Stokes, B.H. *et al*. 2019. Covalent *Plasmodium falciparum*-selective proteasome inhibitors exhibit a low propensity for generating resistance in vitro and synergize with multiple antimalarial agents. PLoS Pathog 15:e1007722. PMID: 31170268.
Cam3.II Kelch13^{C580Y}	Cam3.II^{C580Y}	Zinc Finger Nuclease-mediated editing	Straimer, J. *et al*. 2015. K13-propeller mutations confer artemisinin resistance in *Plasmodium falciparum* clinical isolates. Science 347:428-31. PMID: 25502314.
	Cam3.II K13^{C580Y}	As from Straimer, J. *et al.* 2015	Stokes, B.H. *et al*. 2019. Covalent *Plasmodium falciparum*-selective proteasome inhibitors exhibit a low propensity for generating resistance in vitro and synergize with multiple antimalarial agents. PLoS Pathog 15:e1007722. PMID: 31170268.
Cam3.II Kelch13^{C580Y} Rpt5^{G319S}	Cam3.II K13^{C580Y} RPT5^{G319S}	In vitro selection with WLW	Stokes, B.H. *et al*. 2019. Covalent *Plasmodium falciparum*-selective proteasome inhibitors exhibit a low propensity for generating resistance in vitro and synergize with multiple antimalarial agents. PLoS Pathog 15:e1007722. PMID: 31170268.
Cam3.II Kelch13^{C580Y} β2^{C31F}	Cam3.II K13^{C580Y} β2 C31F	In vitro selection with WLW	Stokes, B.H. *et al*. 2019. Covalent *Plasmodium falciparum*-selective proteasome inhibitors exhibit a low propensity for generating resistance in vitro and synergize with multiple antimalarial agents. PLoS Pathog 15:e1007722. PMID: 31170268.
Cam3.II Kelch13^{C580Y} β2^{C31Y}	Cam3.II K13^{C580Y} β2 C31Y	In vitro selection with WLW	Stokes, B.H. *et al*. 2019. Covalent *Plasmodium falciparum*-selective proteasome inhibitors exhibit a low propensity for generating resistance in vitro and synergize with multiple antimalarial agents. PLoS Pathog 15:e1007722. PMID: 31170268.
Cam3.II Kelch13^{C580Y} β5^{A20S}	Cam3.II K13^{C580Y} β5 A20S	In vitro selection with WLL	Stokes, B.H. *et al*. 2019. Covalent *Plasmodium falciparum*-selective proteasome inhibitors exhibit a low propensity for generating resistance in vitro and synergize with multiple antimalarial agents. PLoS Pathog 15:e1007722. PMID: 31170268.

Table S1: Parasite strains used in this study. Parasite strains are annotated with the name used in this study, any previous names, and how the parasites were obtained.
